# Supplementary figures and images for: Photodamage repair pathways contribute to the accurate maintenance of the DNA methylome landscape upon UV exposure
Source: PLoS Genet. 2019 Nov 18;15(11):e1008476. doi: 10.1371/journal.pgen.1008476 (PMC6886878; doi:10.1371/journal.pgen.1008476)

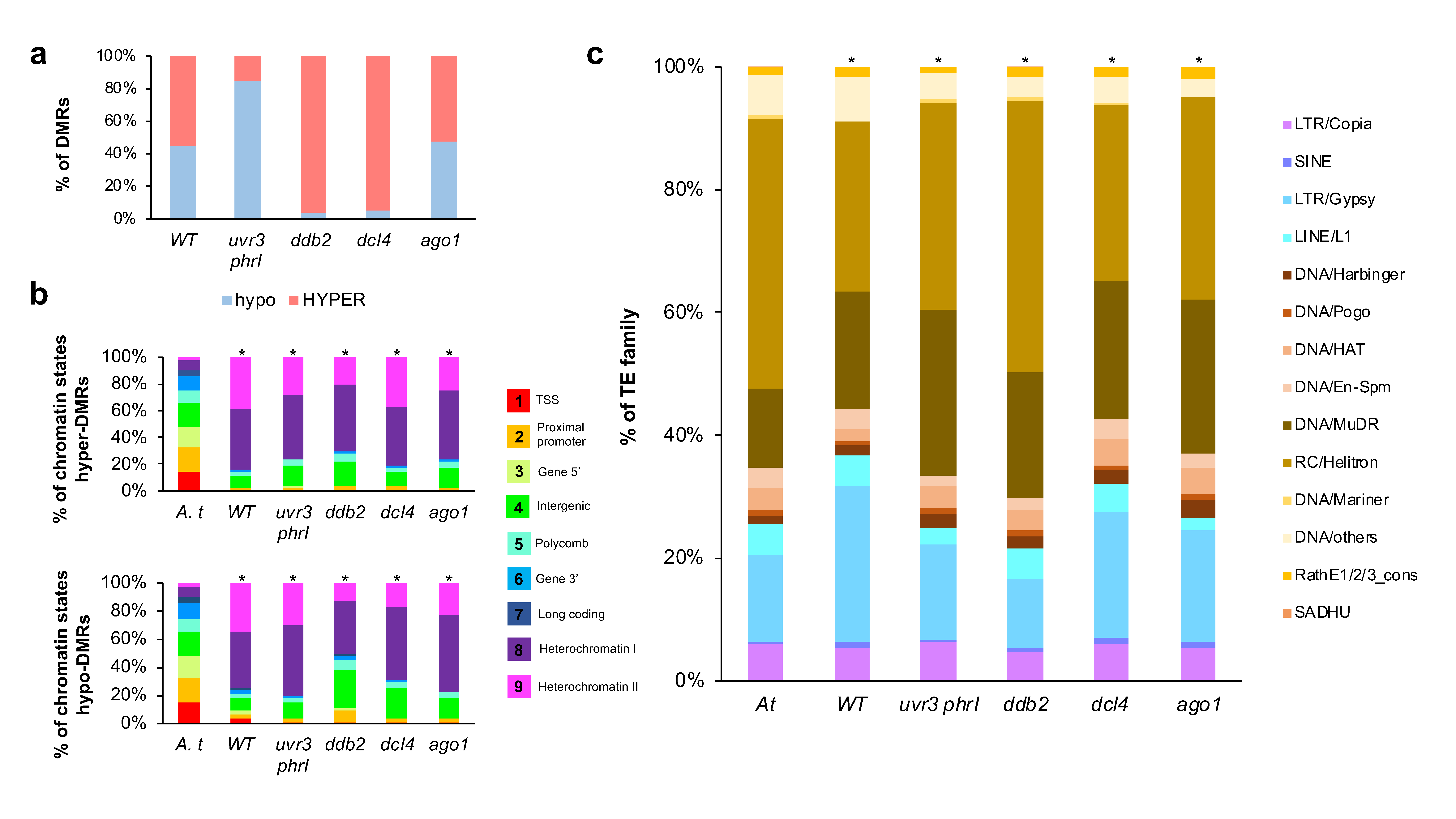

Supplement: S1 Fig — a Histograms representing the percentage of hypo-DMRs (red) and hyper-DMRs (blue) identified in WT, uvr3 phrI, ddb2, dcl4 and ago1 plants 24h upon UV-C exposure. b Histograms representing the distribution of the chromatin states overlapping with DMRs identified in WT, uvr3 phrI, ddb2, dcl4 and ago1 plants. A. t represents the overall distribution of the 9 chromatin states in the Arabidopsis genome (A. t). * Chi square test < 0.01 compared to the Arabidopsis genome. c Histograms representing the distribution of TE families overlapping with DMRs identified in WT, uvr3 phrI, ddb2, dcl4 and ago1 plants. A. t represents the overall distribution of the TE families in the Arabidopsis genome (A. t). * Chi square test < 0.01. compared to the Arabidopsis genome. (TIFF) [file pgen.1008476.s001.tiff]

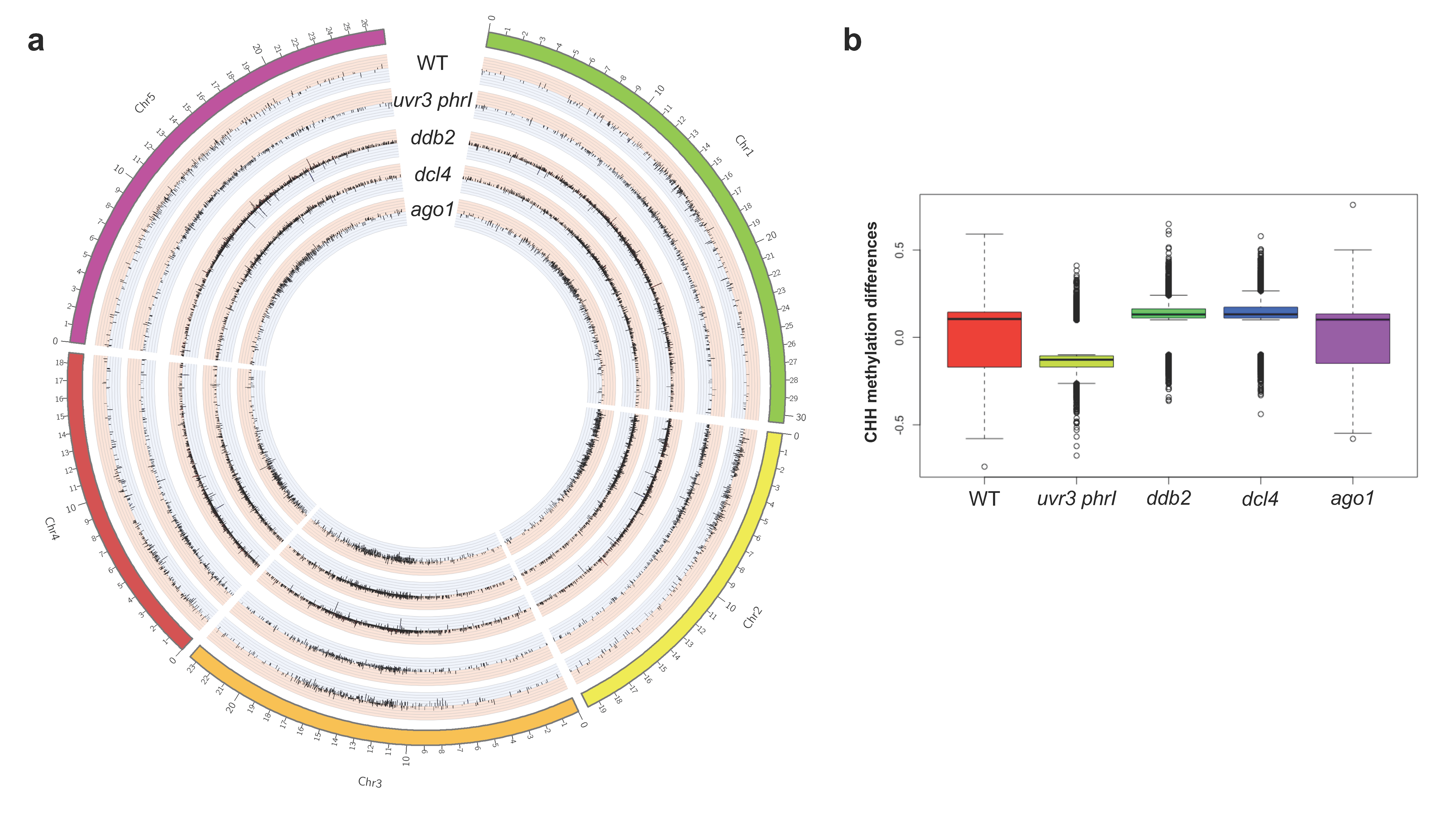

Supplement: S2 Fig — a Circos representation of the DMRs identified in WT, uvr3 phrI, ddb2, dcl4 and ago1 plants 24h upon UV-C exposure. b Boxplots representing the CHH methylation changes in WT, uvr3 phrI, ddb2, dcl4 and ago1 plants 24h upon UV-C exposure. (TIFF) [file pgen.1008476.s002.tiff]

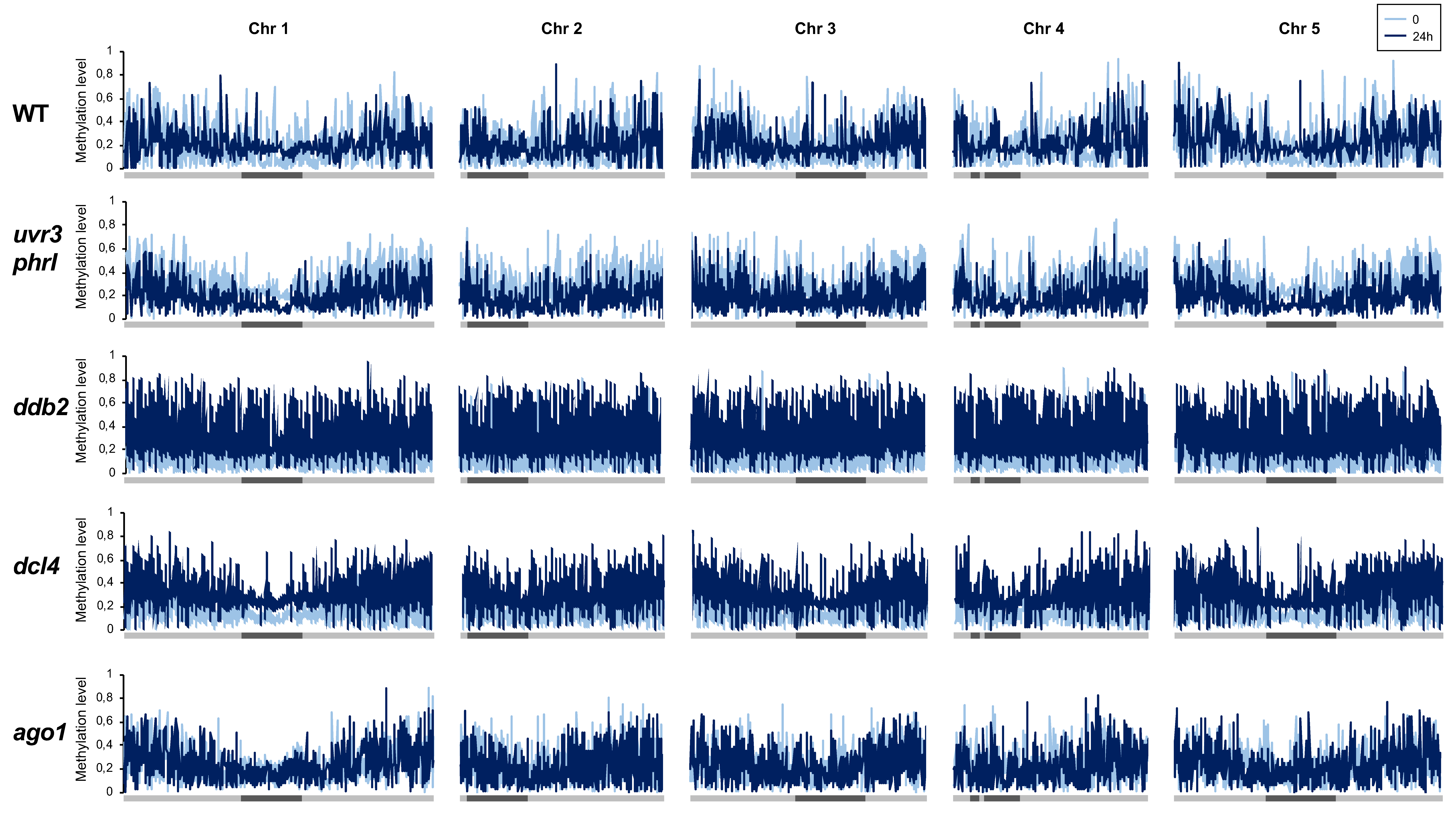

Supplement: S3 Fig — CHH DNA methylation levels along chromosomes(light grey: chromosome arms, dark gray: pericentromeric regions) in WT, uvr3 phrI, ddb2, dcl4 and ago1 plants prior (0) and 24h upon UV-C exposure. (TIFF) [file pgen.1008476.s003.tiff]

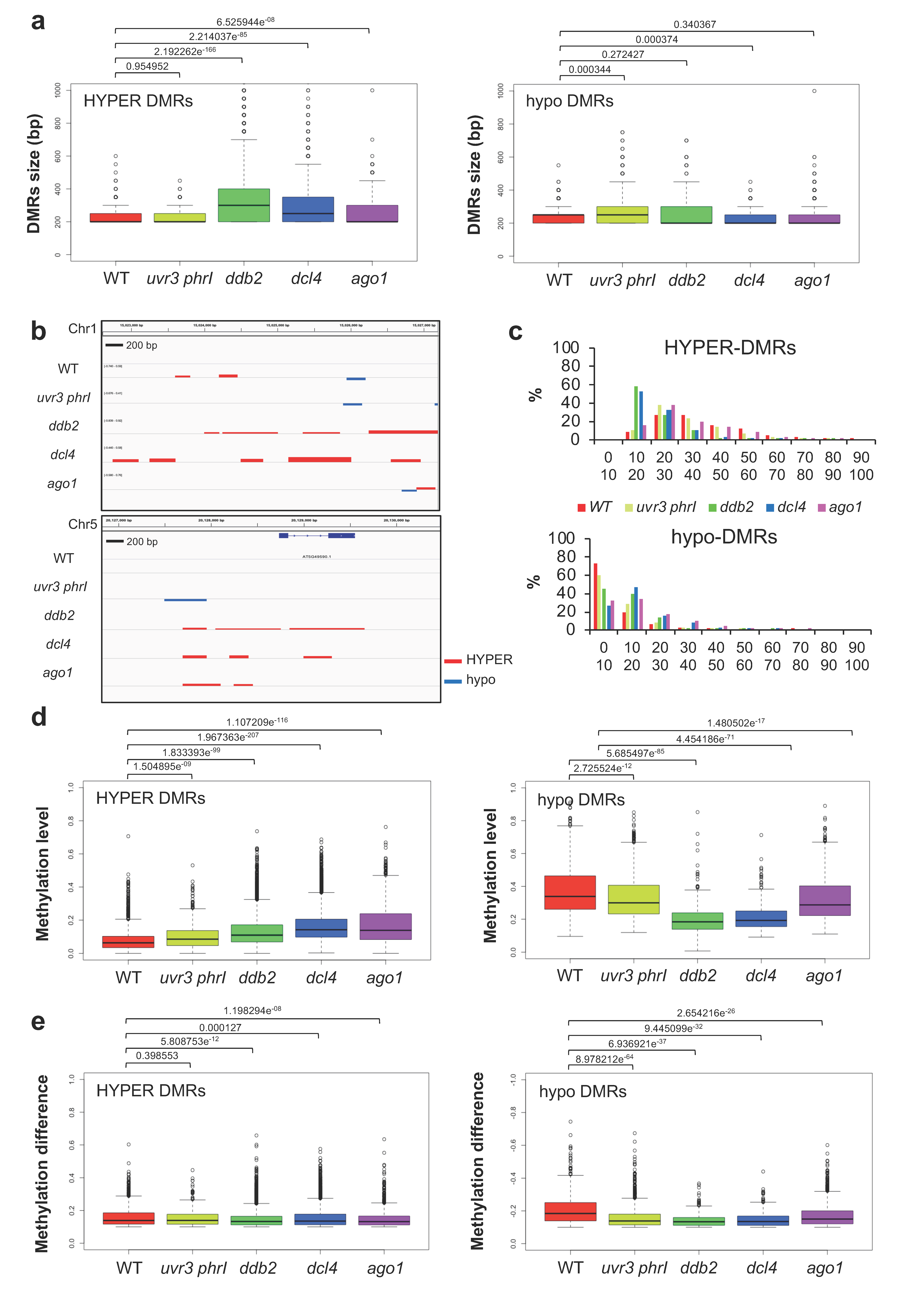

Supplement: S4 Fig — a Boxplots representing the size (bp) of hyper-DMRs (left panel) and hypo-DMRs (right panel) identified in WT, uvr3 phrI, ddb2, dcl4 and ago1 plants. Exact p values according Mann Whitney test are indicated above each graph. b Genome browser views of hyper- and hypo-DMRs identified in WT, uvr3 phrI, ddb2, dcl4 and ago1 plants upon UV-C exposure. Upper panel: Chr1: 15, 023, 000 bp-15, 027, 000 bp intergenic region. Lower panel: Chr5: 20, 127, 000 bp-20, 130, 000 bp intergenic region/PCG. Red line: HYPER-DMRs; blue line: hyo-DMRs. c Histograms representing the distribution of methylation levels prior UV-C irradiation in genomic regions exhibiting hypo- (left panel) and hyper-DMRs (right panel). d Boxplots representing the CHH methylation levels prior UV-C irradiation in genomic regions exhibiting hypo- (left panel) and hyper-DMRs (right panel) identified in WT, uvr3 phrI, ddb2, dcl4 and ago1 plants. Exact p values according Mann Whitney test are indicated above each graph. e Boxplots representing the CHH methylation difference of hypo- (left panel) and hyper-DMRs (right panel) identified in WT, uvr3 phrI, ddb2, dcl4 and ago1 plants 24h upon UV-C exposure. Exact p values according Mann Whitney test are indicated above each graph. (TIFF) [file pgen.1008476.s004.tiff]

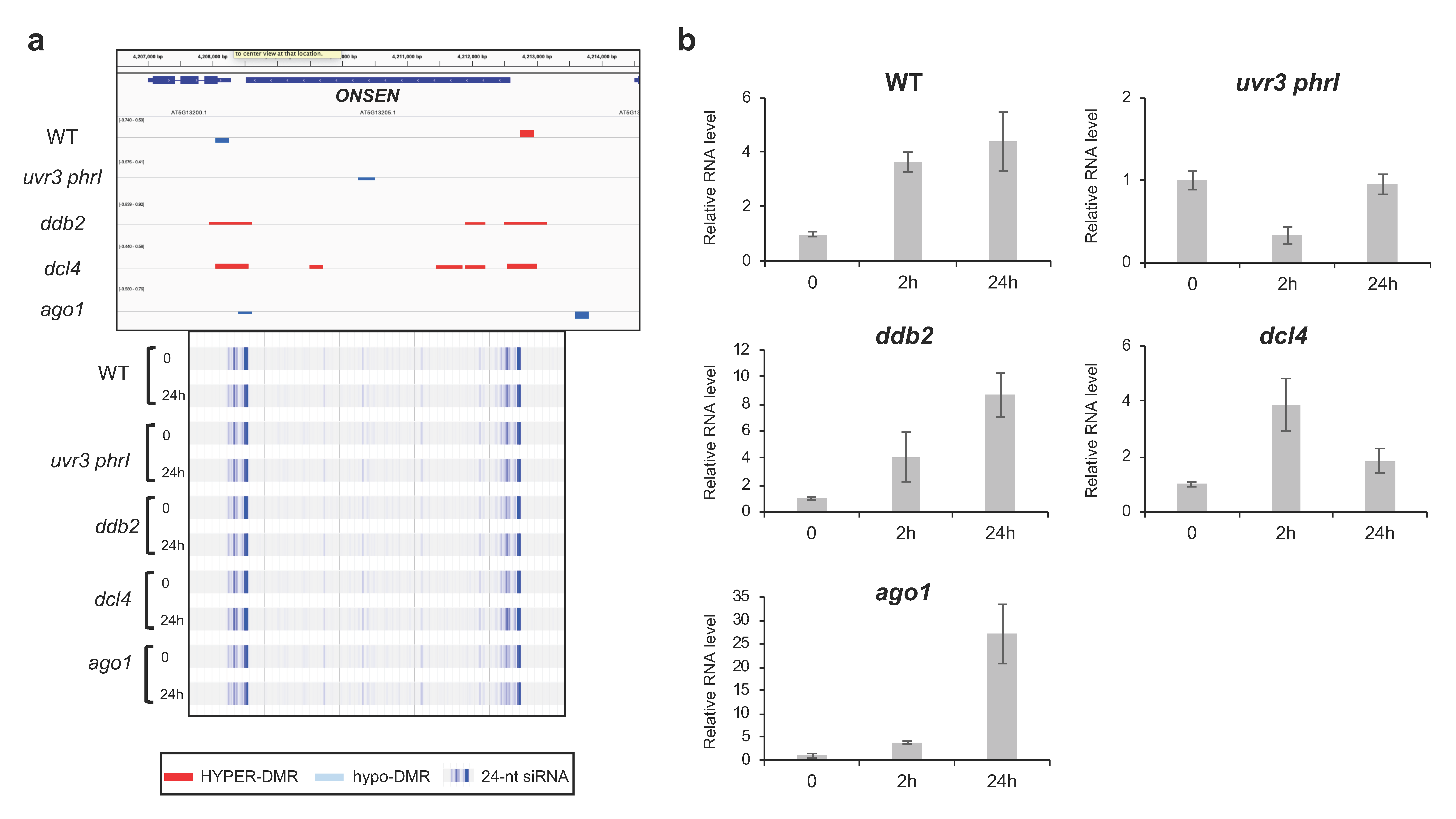

Supplement: S5 Fig — a Genome browser showing the density graph 24-nt siRNA abundance and DMRs at ONSEN locus in WT, uvr3 phrI, ddb2, dcl4 and ago1 plants prior UV-C irradiation (0) and upon UV-C exposure (24h). b RNA steady state level of ONSEN transcripts determined by RT-qPCR in WT, uvr3 phrI, ddb2, dcl4 and ago1 plants before (0), 2h and 24h following UV-C irradiation. (TIFF) [file pgen.1008476.s005.tiff]

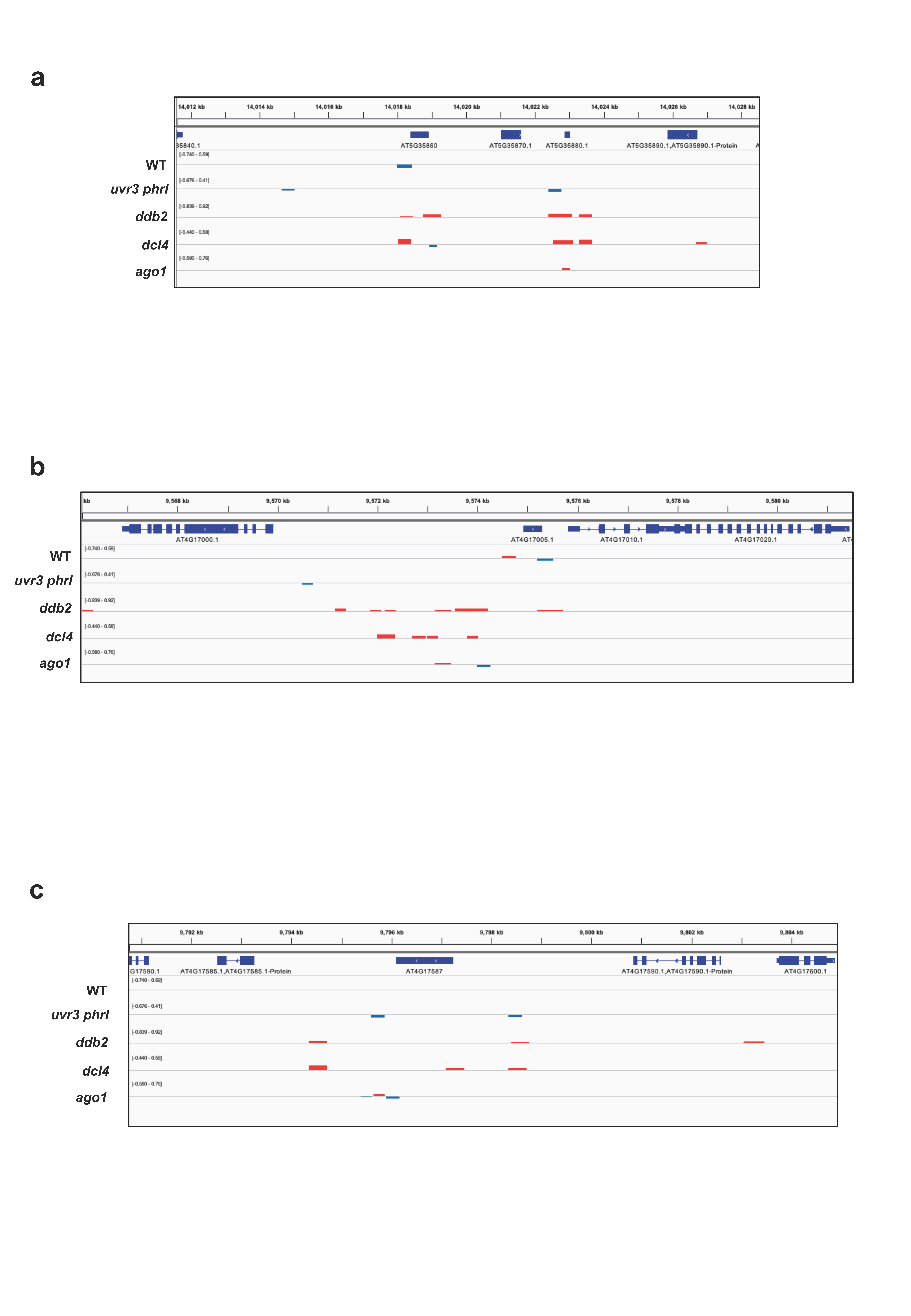

Supplement: S6 Fig — Examples of hyper- and hypo-DMRs identified in WT, uvr3 phrI, ddb2, dcl4 and ago1 plants upon UV-C exposure in TE enriched region (a) and in intergenic regions surrounding protein coding genes (b) or TE (c). (TIFF) [file pgen.1008476.s006.tiff]

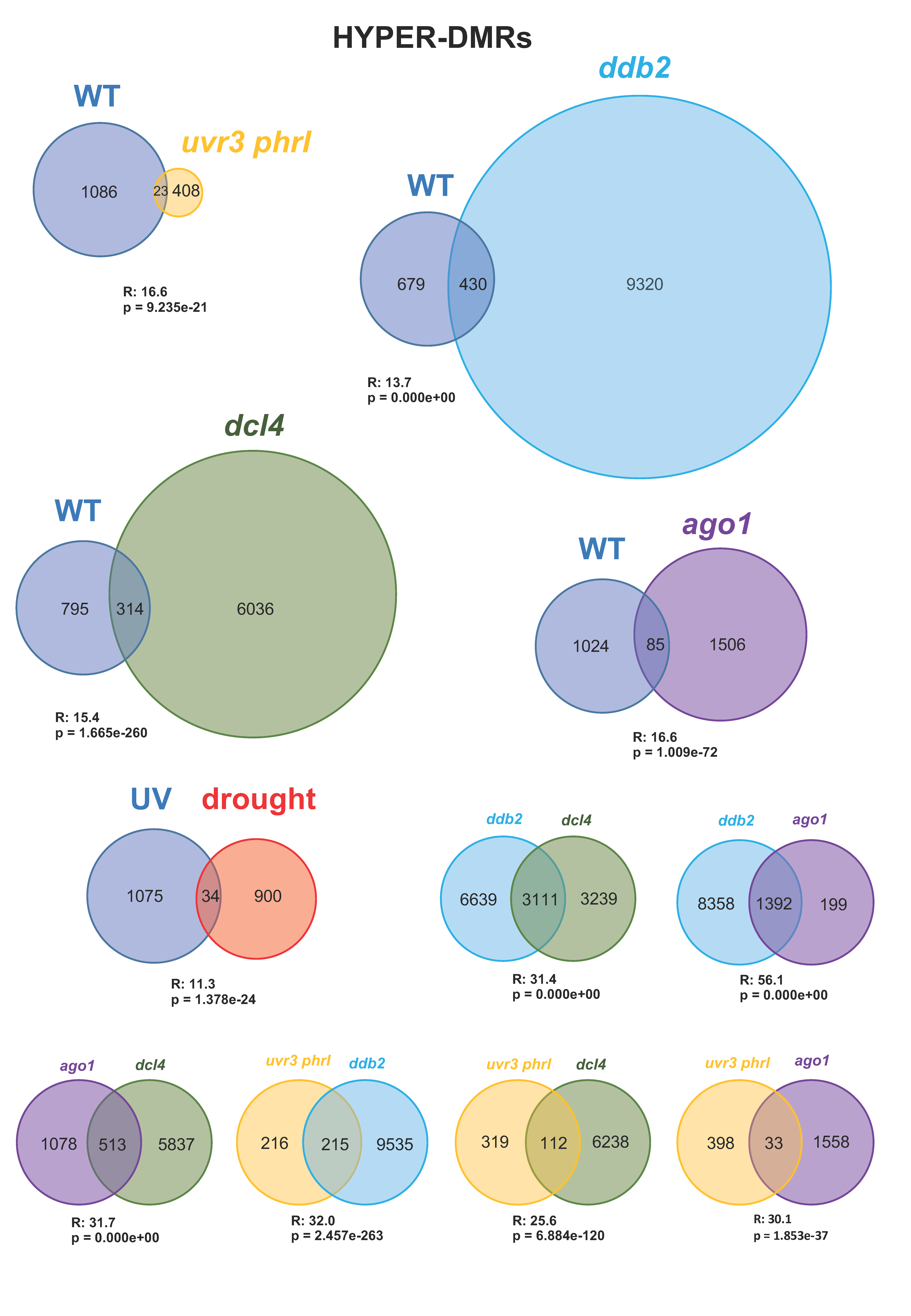

Supplement: S7 Fig — Venn diagrams representing the overlap of hyper-DMRs between WT plants and either uvr3 phrI, ddb2, dcl4, ago1 or WT drought plants as well as in between mutant plants. R: Representation factor and exact p value showing the statistical significance of the overlap. (TIFF) [file pgen.1008476.s007.tiff]

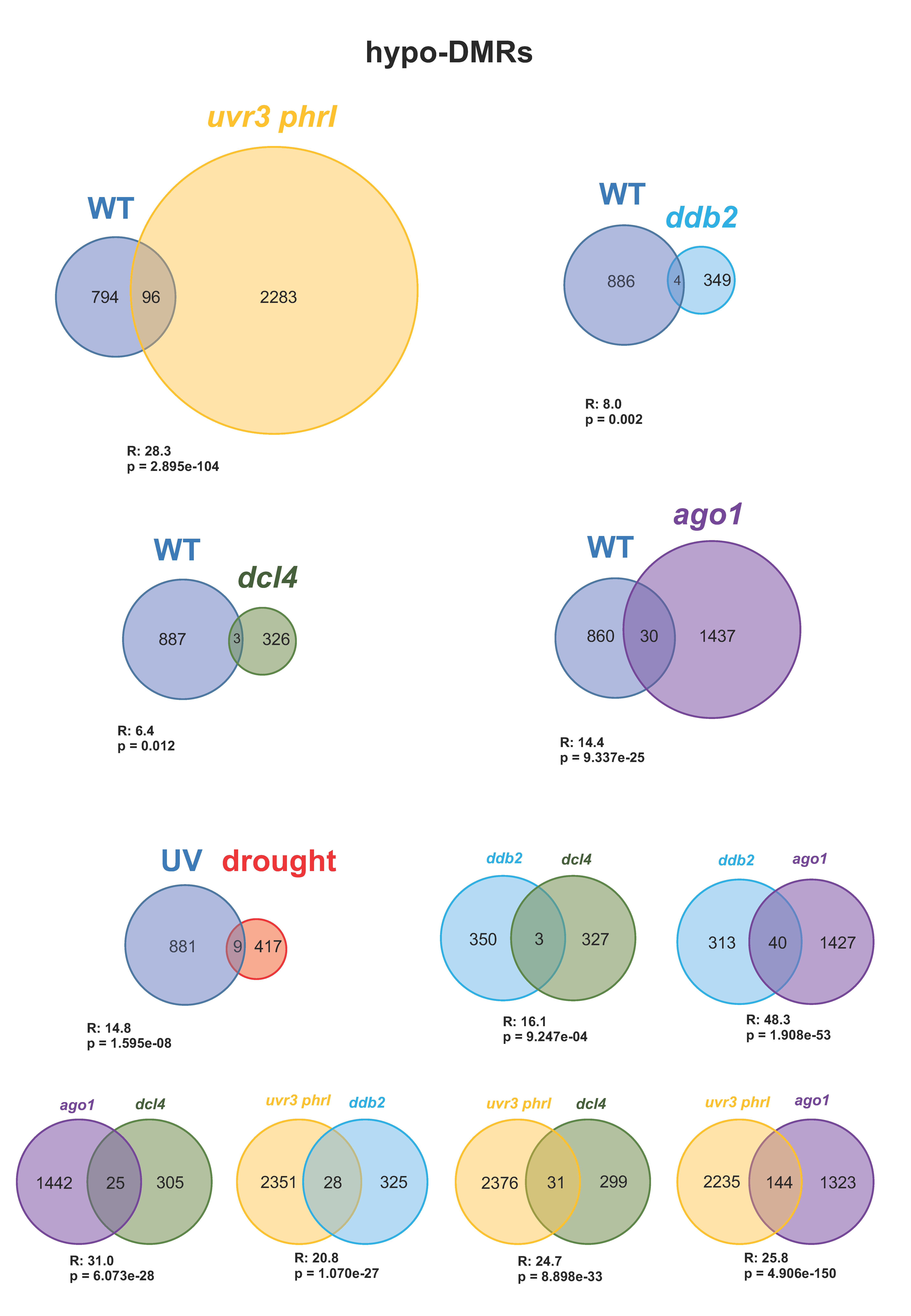

Supplement: S8 Fig — Venn diagrams representing the overlap of hypo-DMRs between WT plants and either uvr3 phrI, ddb2, dcl4, ago1 or WT drought plants as well as in between mutant plants. R: Representation factor and exact p value showing the statistical significance of the overlap. (TIFF) [file pgen.1008476.s008.tiff]

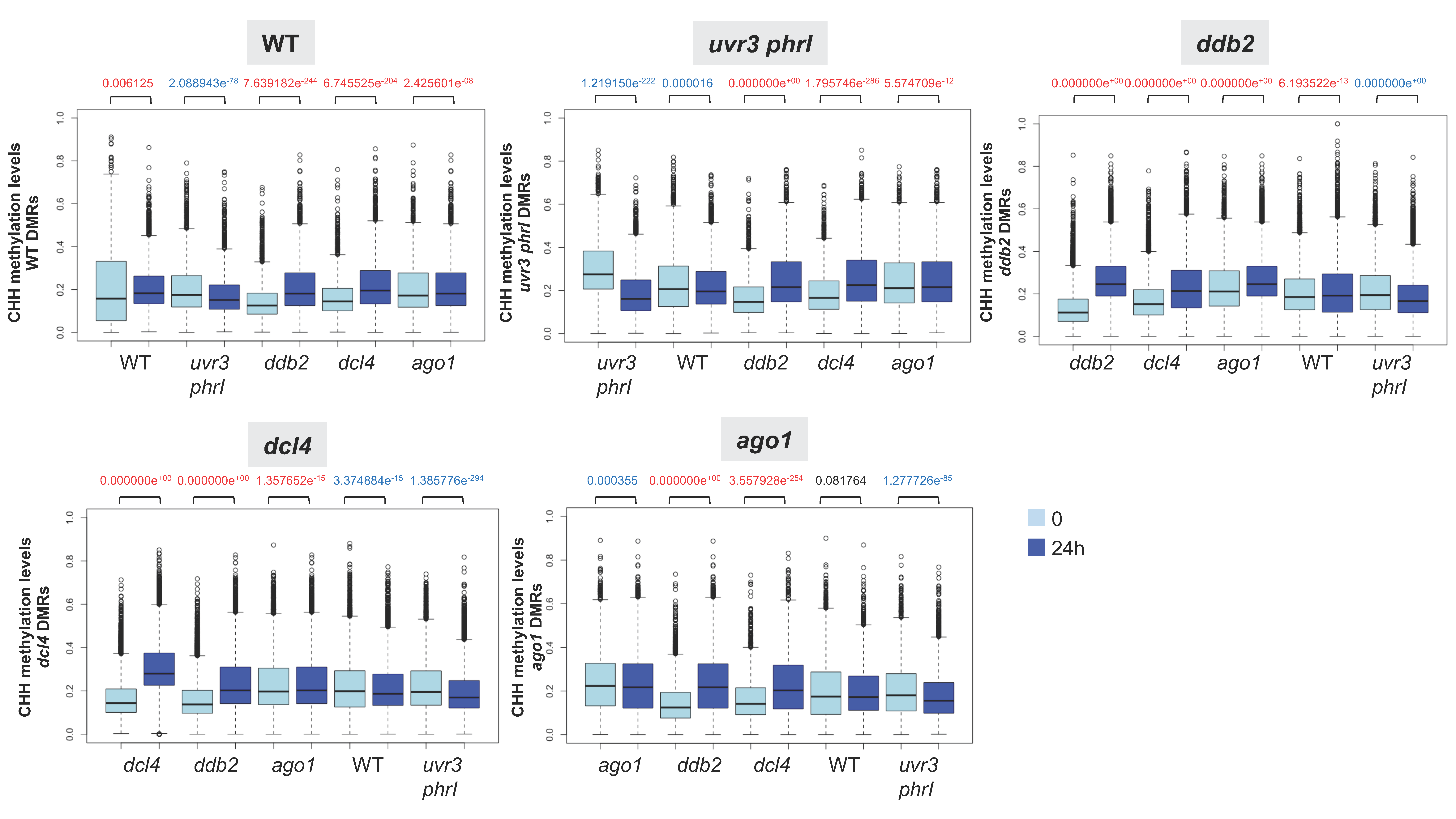

Supplement: S9 Fig — Boxplot of CHH methylation levels within DMRs identified in WT, uvr3 phrI, ddb2, dcl4 and ago1 plants 24h upon UV-C exposure. The CHH methylation levels of each of these DMRs are reported before and upon UV-C exposure for each plant. Exact p values according Wilcoxon signed rank test are indicated above each graph. Blue significant decrease of DNA methylation level, red significant increase of DNA methylation level, black non-significant change. (TIFF) [file pgen.1008476.s009.tiff]

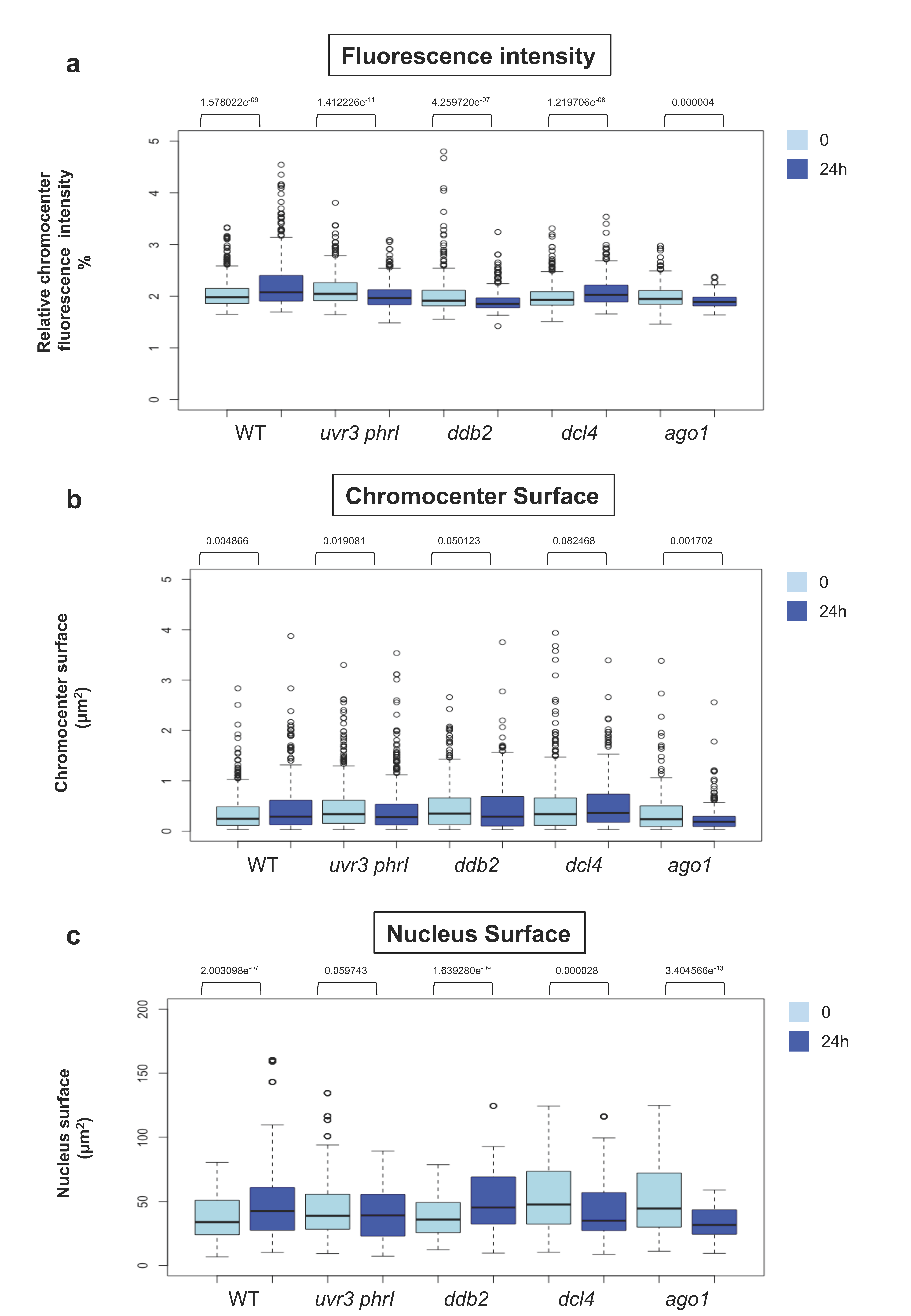

Supplement: S10 Fig — Boxplots representing the relative fluorescence intensities of chromocenters (a), chromocenter surface (b) and nucleus surface (c) of untreated (time point 0) and UV-C treated (time point 24h) WT, uvr3 phrI, ddb2, dcl4 and ago1 plants. Exact p values according Mann Whitney test are indicated above each graph. Number of chromocenters analyzed: 252 to 601; Number of nuclei analyzed: 44 to 109. (TIFF) [file pgen.1008476.s010.tiff]

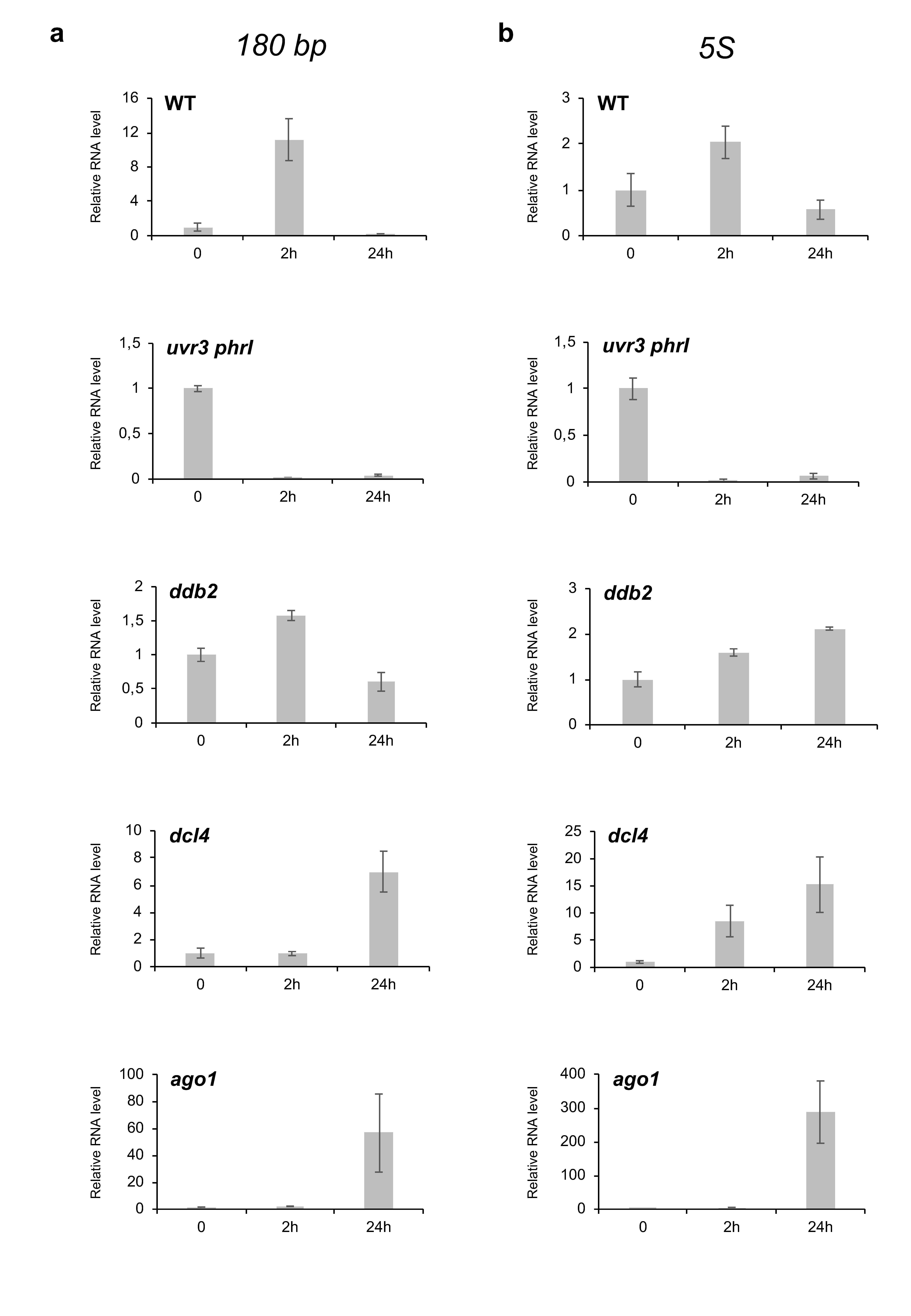

Supplement: S11 Fig — Relative RNA steady state level (±SD) of 180 bp (a) and 5S RNA (b) transcripts determined by RT-qPCR in WT, uvr3 phrI, ddb2, dcl4 and ago1 plants before (0), 2h and 24h following UV-C irradiation. (TIFF) [file pgen.1008476.s011.tiff]

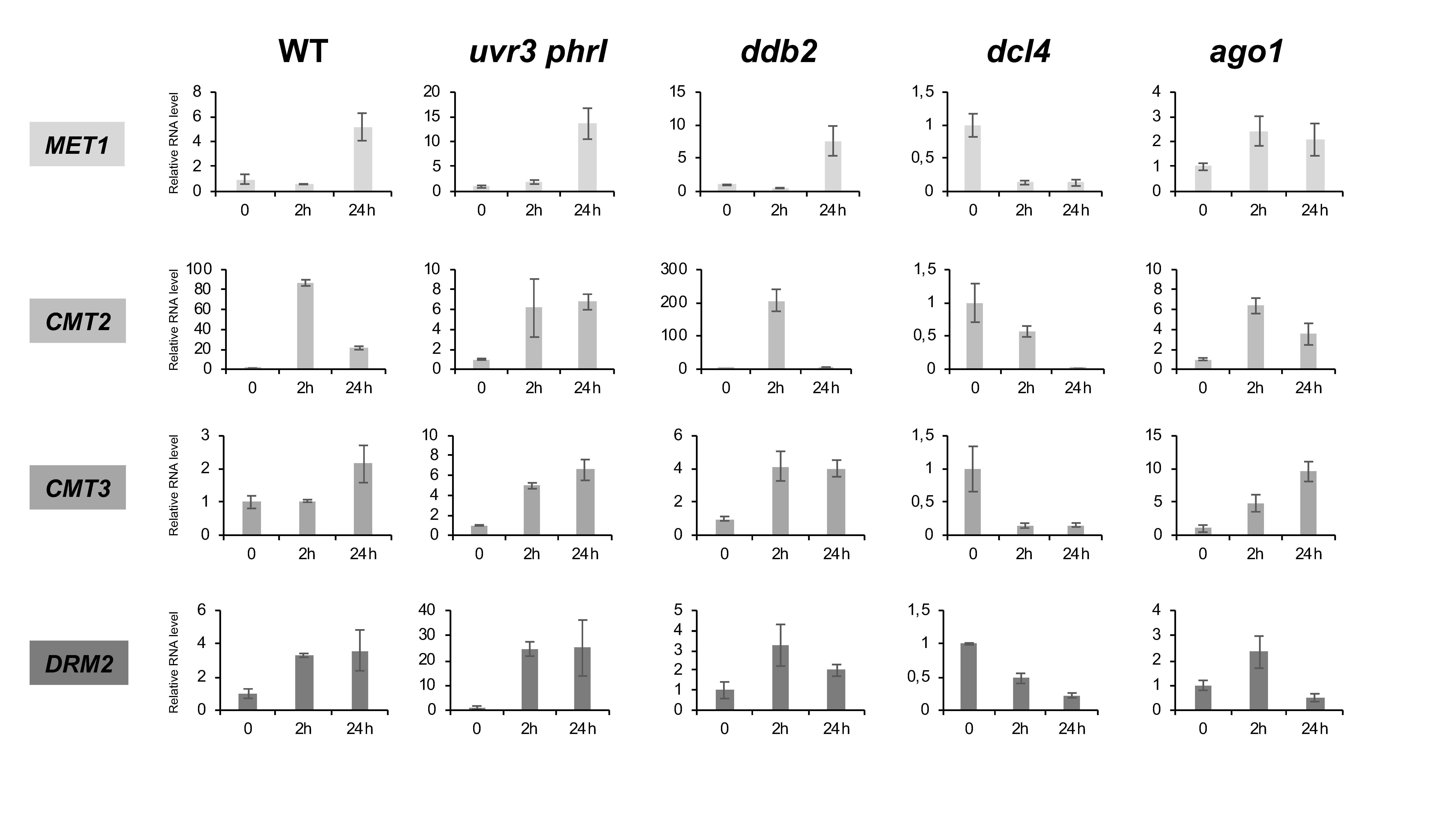

Supplement: S12 Fig — Relative RNA steady state level (±SD) of MET1, CMT2, CMT3 and DRM2 transcripts determined by RT-qPCR in WT, uvr3 phrI, ddb2, dcl4 and ago1 plants before (0), 2h and 24h following UV-C irradiation. (TIFF) [file pgen.1008476.s012.tiff]

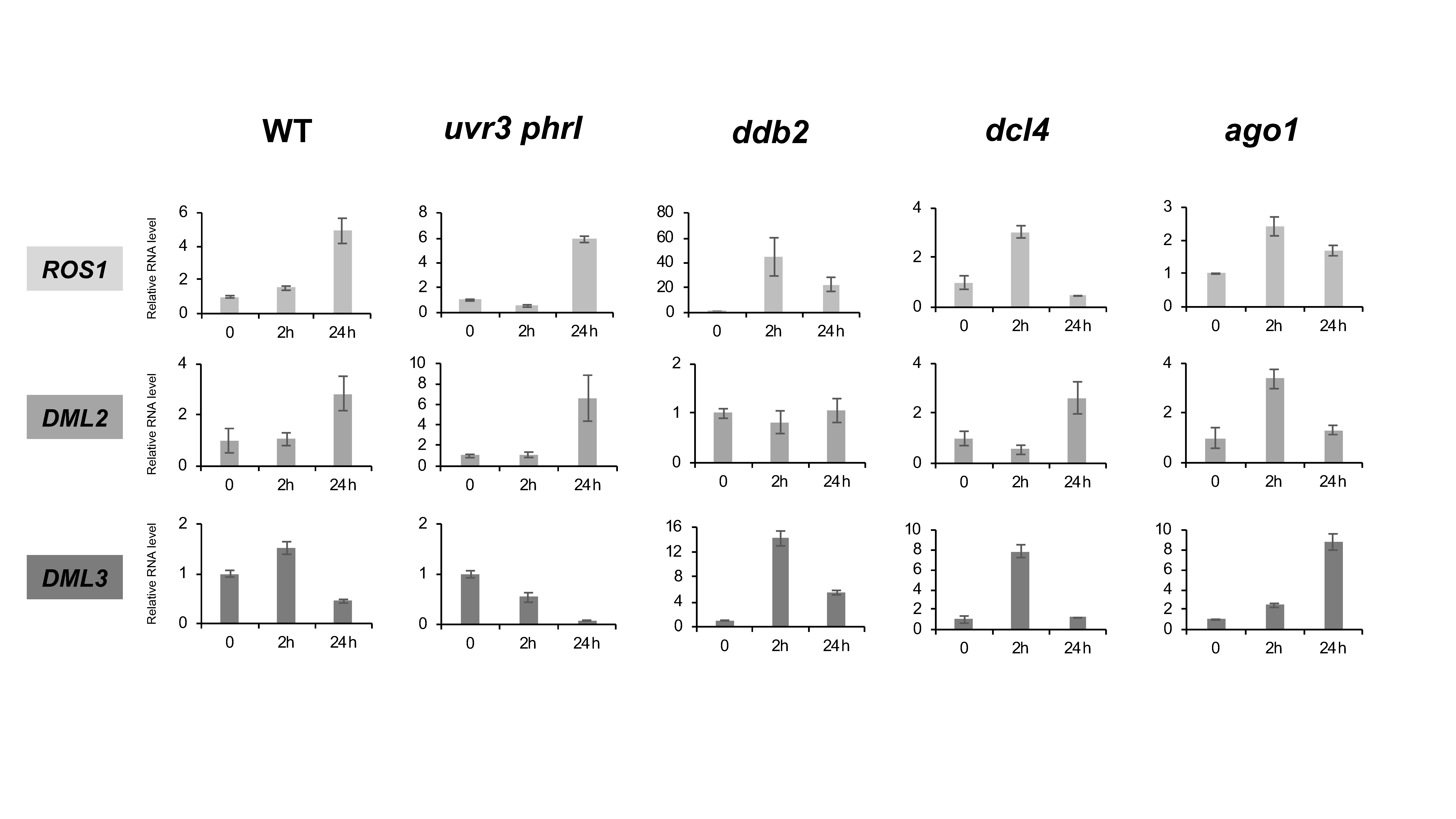

Supplement: S13 Fig — Relative RNA steady state level (±SD) of ROS1, DML2 and DML3 transcripts determined by RT-qPCR in WT, uvr3 phrI, ddb2, dcl4 and ago1 plants before (0), 2h and 24h following UV-C irradiation. (TIFF) [file pgen.1008476.s013.tiff]

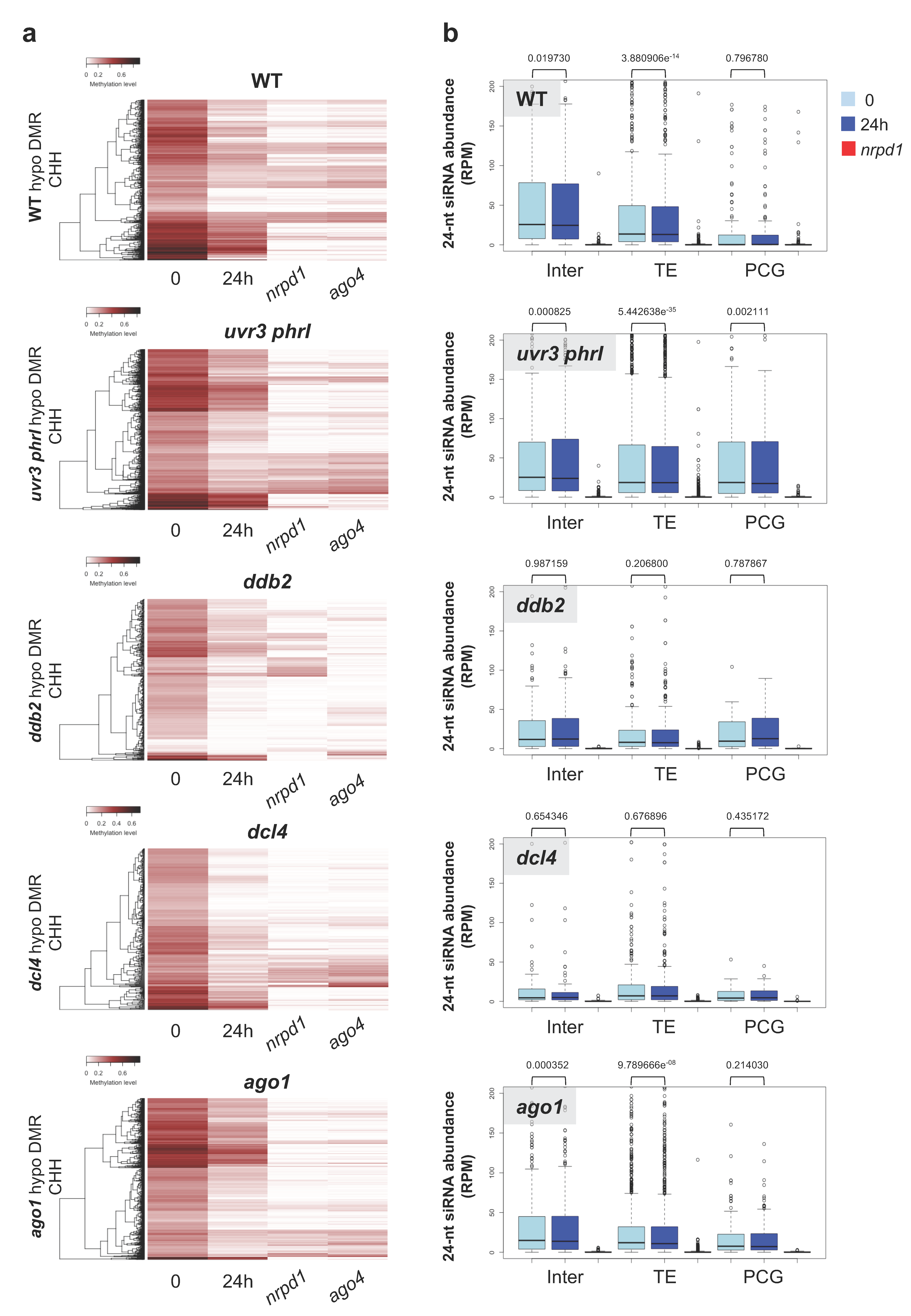

Supplement: S14 Fig — a Heatmaps of CHH methylation levels within hypo-DMRs identified in WT, uvr3 phrI, ddb2, dcl4 and ago1 plants before, 24h upon UV-C exposure. The CHH methylation levels of each of these hypo-DMR are reported for RNA POL IV (nrpd1) and AGO4 (ago4) deficient plants. (white: 0; black: 1). b Boxplots representing the abundance of 24-nt siRNAs mapping to the CHH hypo-DMRs identified in WT, uvr3 phrI, ddb2, dcl4, ago1 plants for protein-coding genes (PCG), TE and intergenic regions. For each genotype the abundance of 24-nt siRNAs is shown in RNA POL IV deficient plants (nrpd1). The 24-nt siRNA abundance is normalized against global small RNA content and expressed as reads per million (RPM). p-values are calculated according to Wilcoxon Matched-Pairs Signed-Ranks. (TIFF) [file pgen.1008476.s014.tiff]

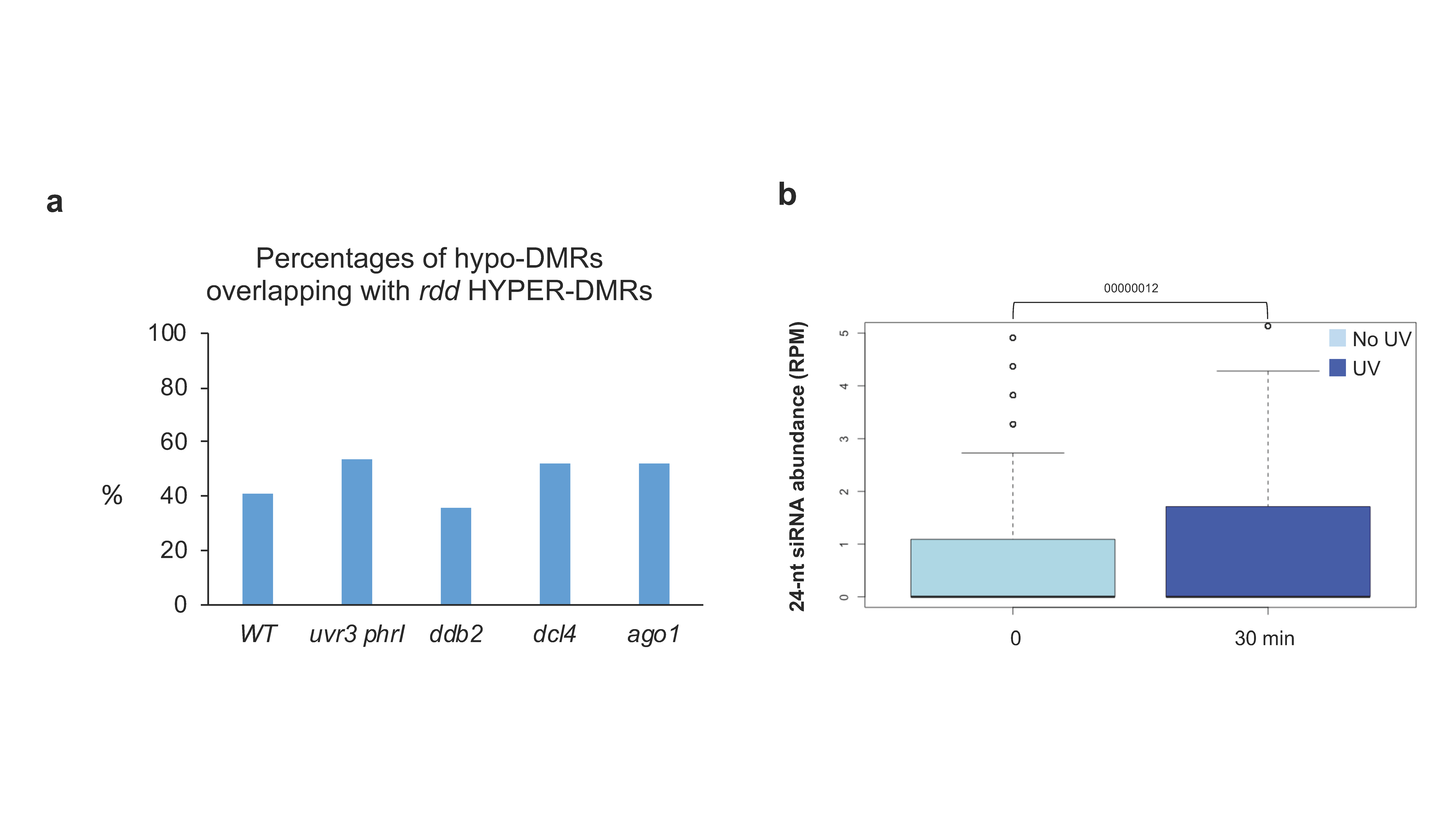

Supplement: S15 Fig — a Histograms representing the percentages of hypo-DMRs identified in WT, uvr3 phrI, ddb2, dcl4, ago1 plants overlapping with rdd hyper-DMRs. b Boxplots representing the global 24-nt siRNA abundance before and 30 min following UV-C exposure. p-value is calculated according to Wilcoxon Matched-Pairs Signed-Ranks. (TIFF) [file pgen.1008476.s015.tiff]

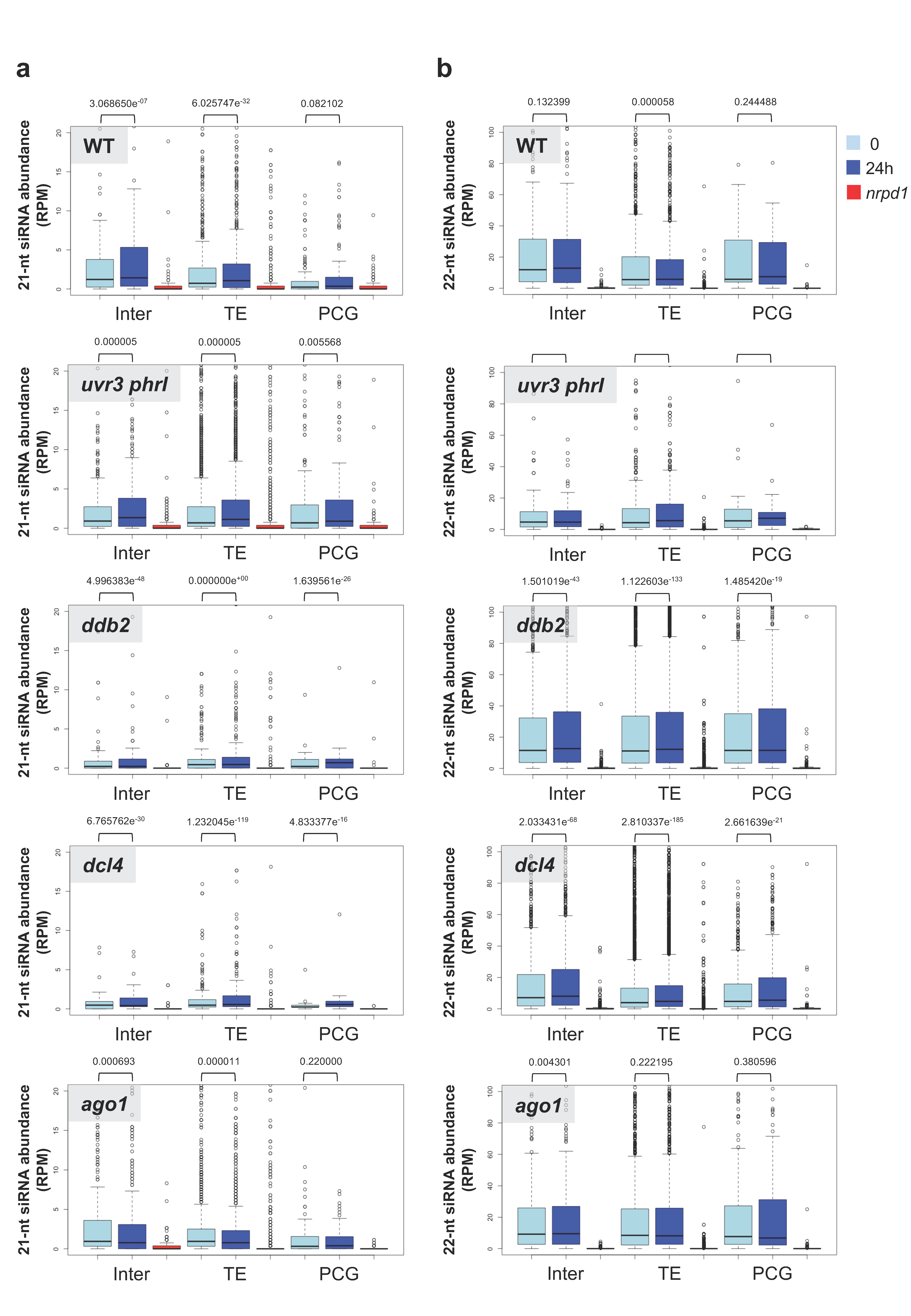

Supplement: S16 Fig — Boxplots representing the abundance of 21-nt (a) and 22-nt (b) siRNAs mapping to the CHH hyper-DMRs identified in WT, uvr3 phrI, ddb2, dcl4, ago1 plants for protein-coding genes (PCG), TE and intergenic regions. For each genotype the abundance of 21-nt and 22-nt siRNAs is shown in RNA POL IV deficient plants (nrpd1). The 21-nt and 22-nt siRNA abundance are normalized against global small RNA content and expressed as reads per million (RPM). p-values are calculated according to Wilcoxon Matched-Pairs Signed-Ranks. (TIFF) [file pgen.1008476.s016.tiff]

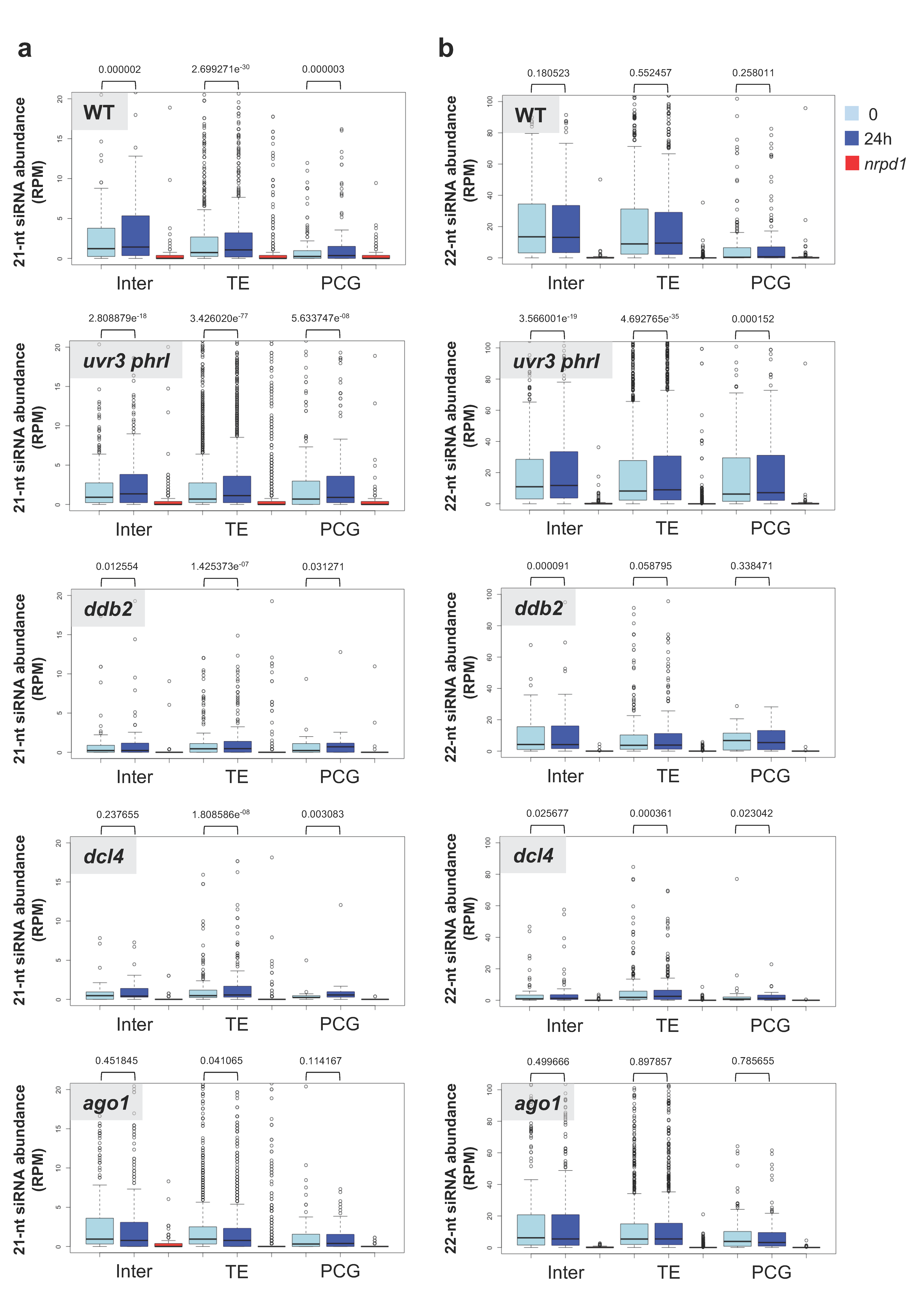

Supplement: S17 Fig — Boxplots representing the abundance of 21-nt (a) and 22-nt (b) siRNAs mapping to the CHH hypo-DMRs identified in WT, uvr3 phrI, ddb2, dcl4, ago1 plants for protein-coding genes (PCG), TE and intergenic regions. For each genotype the abundance of 21-nt and 22-nt siRNAs is shown in RNA POL IV deficient plants (nrpd1). The 21-nt and 22-nt siRNA abundance are normalized against global small RNA content and expressed as reads per million (RPM). p-values are calculated according to Wilcoxon Matched-Pairs Signed-Ranks. (TIFF) [file pgen.1008476.s017.tiff]

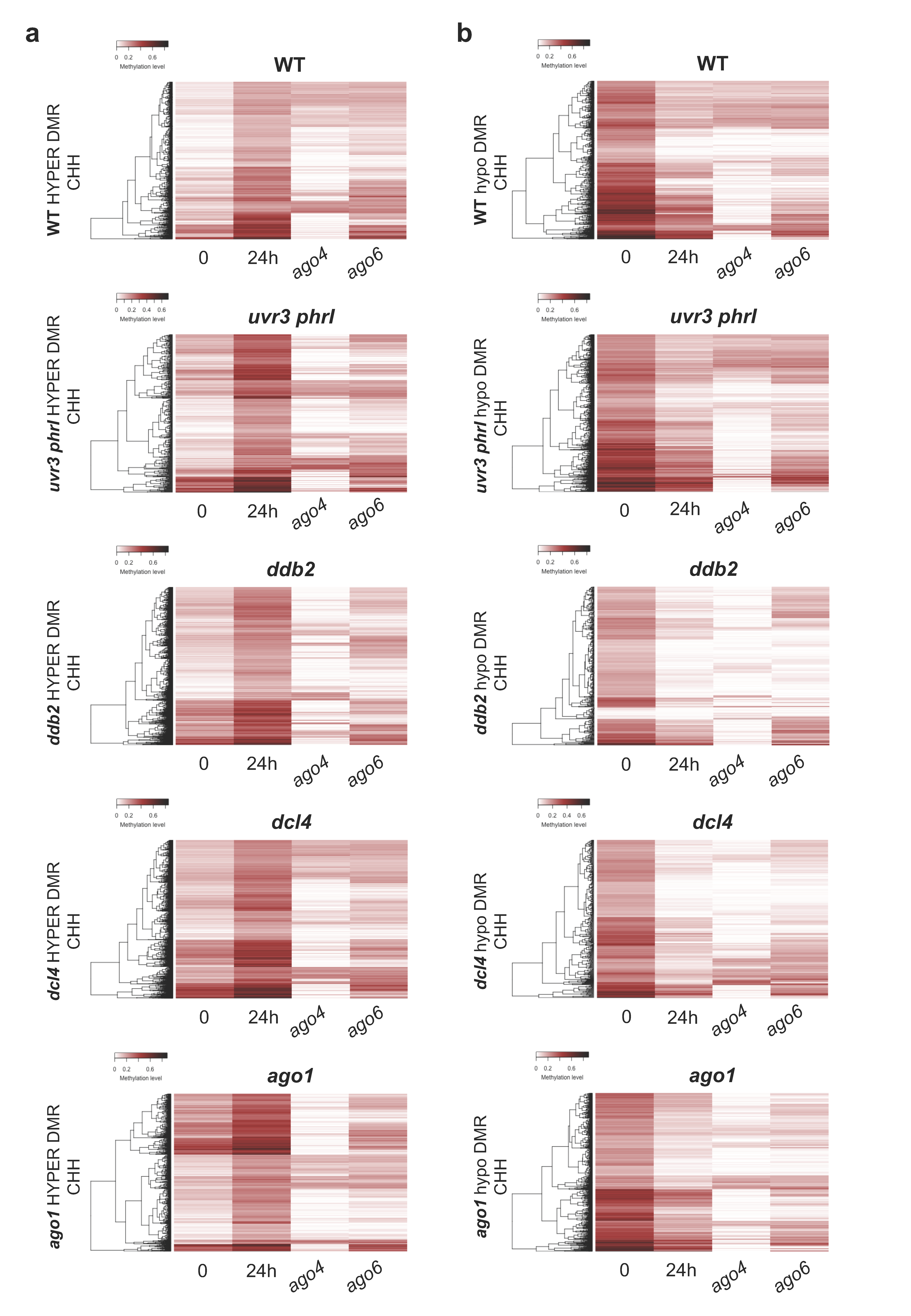

Supplement: S18 Fig — Heatmaps of CHH methylation levels within hyper- (a) and hypo-DMRs (b) identified in WT, uvr3 phrI, ddb2, dcl4 and ago1 plants before and 24h upon UV-C exposure. The CHH methylation levels of each of these DMRs are reported for AGO4 (ago4) and AGO6 (ago6) deficient plants. Columns represent data for each indicated genotype (white: 0; black: 1). (TIFF) [file pgen.1008476.s018.tiff]

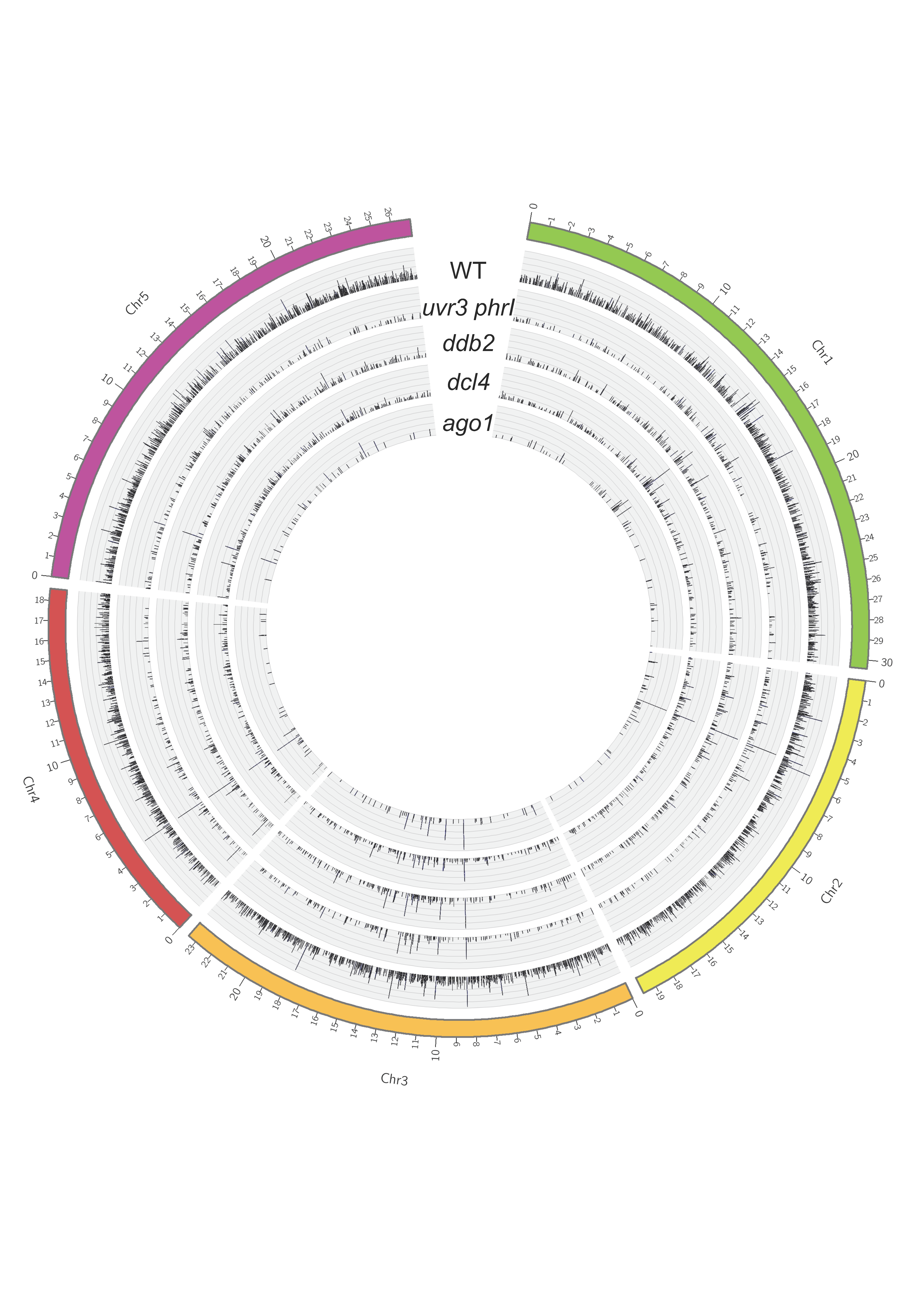

Supplement: S19 Fig — Circos representation of photolesions identified in WT, uvr3 phrI, ddb2, dcl4 and ago1 plants. (TIFF) [file pgen.1008476.s019.tiff]

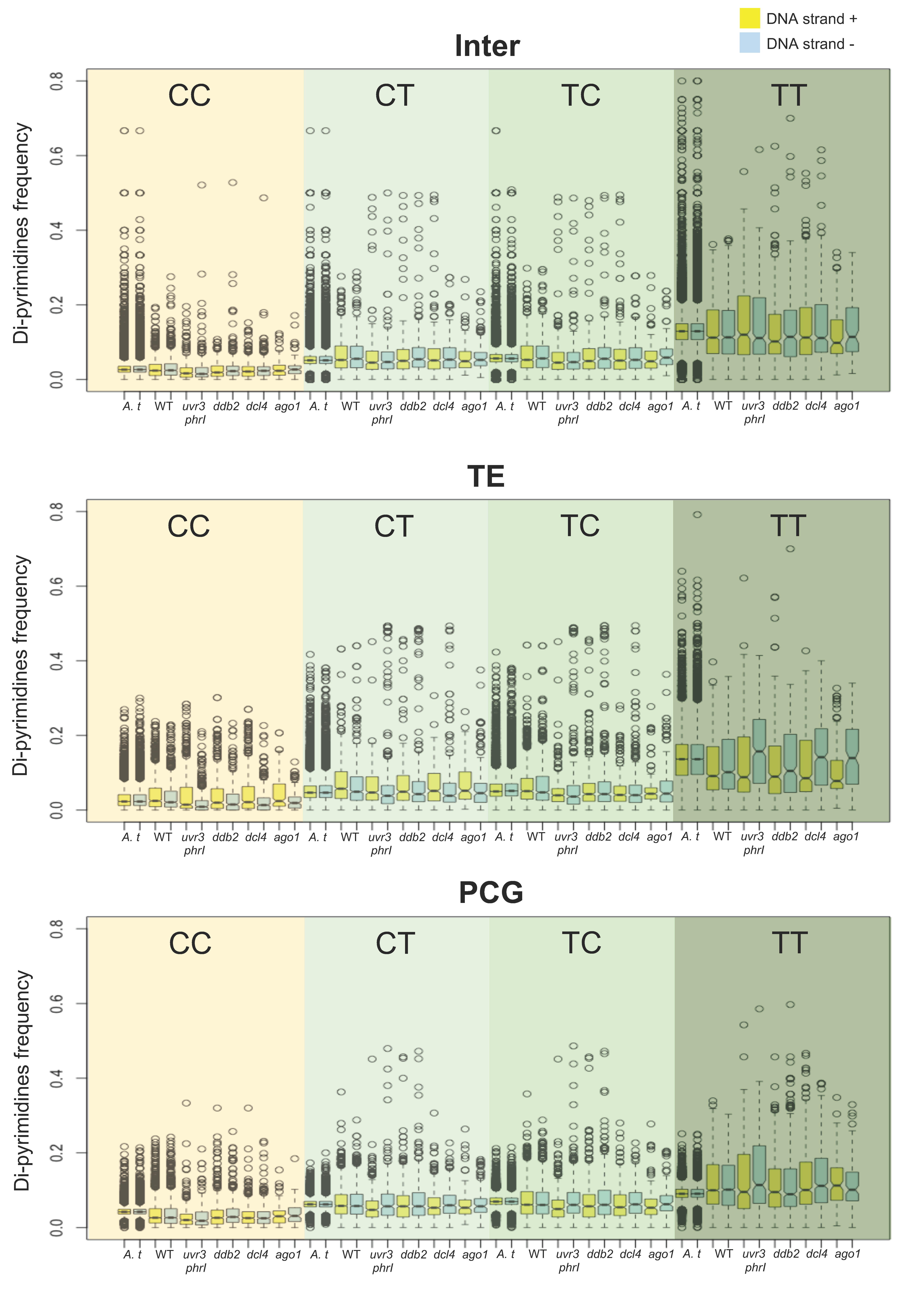

Supplement: S20 Fig — Boxplots representing the di-pyrimidines frequencies (CC, TT, TC and CT) for each DNA strand (+ and–strand) in photodamaged regions (intergenic, TE and protein-coding genes: PCG) identified in WT, uvr3 phrI, ddb2, dcl4 and ago1 plants. The frequency of di-pyrimidine in the Arabidopsis thaliana (A. t) genome is also represented. (TIFF) [file pgen.1008476.s020.tiff]

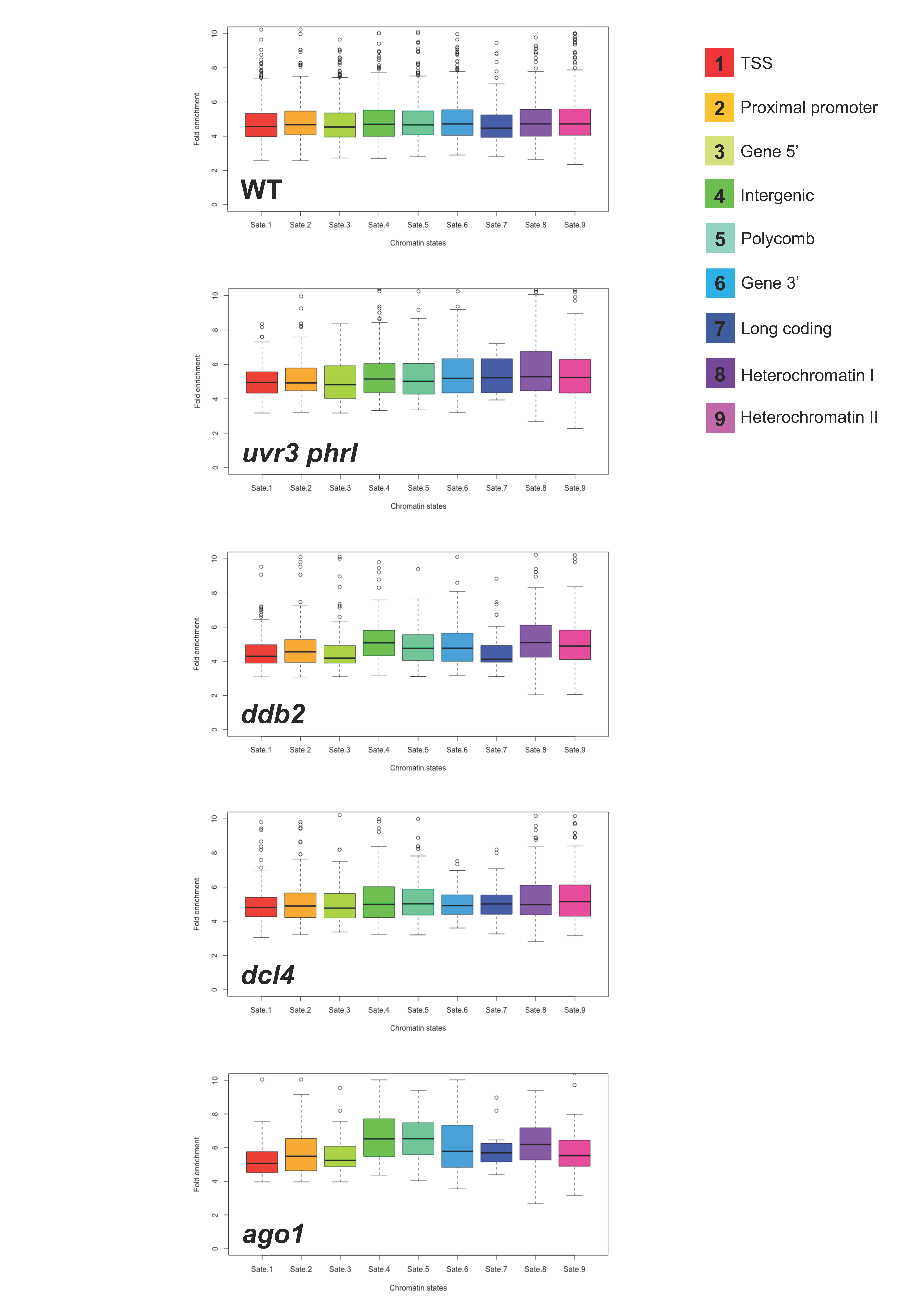

Supplement: S21 Fig — Boxplots representing the chromatin states overlapping with photolesions enriched regions in WT, uvr3 phrI, ddb2, dcl4 and ago1 plants. (TIFF) [file pgen.1008476.s021.tiff]

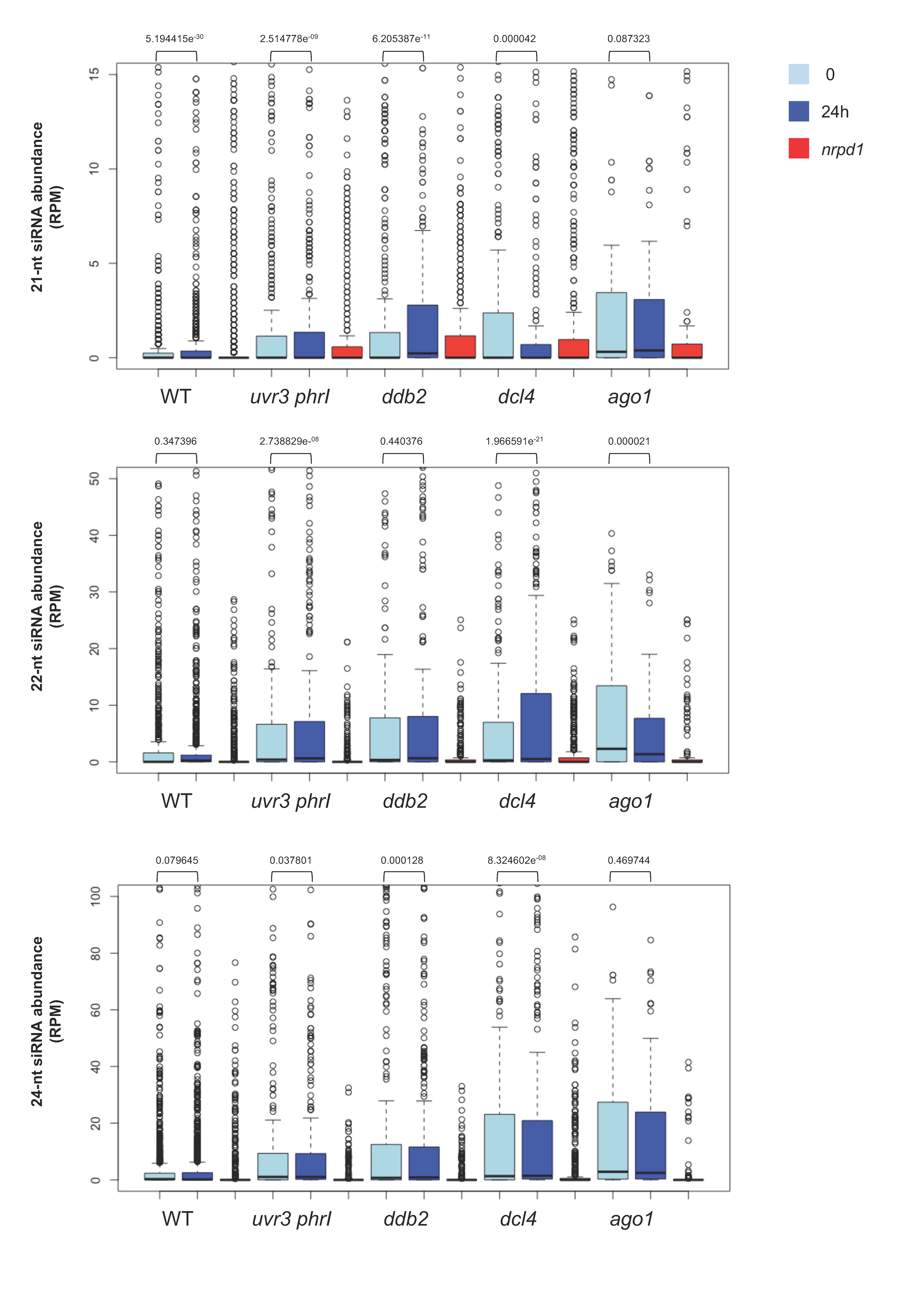

Supplement: S22 Fig — Boxplots representing the abundance of 21-, 22 and 24-nt siRNAs mapping to the photodamaged genomic regions in WT, uvr3 phrI, ddb2, dcl4, ago1 plants. For each genotype the abundance of 21-, 22 and 24-nt siRNAs is shown in RNA POL IV deficient plants (nrpd1). siRNA abundances are normalized against global small RNA content and expressed as reads per million (RPM). p-values are calculated according to Wilcoxon Matched-Pairs Signed-Ranks. (TIFF) [file pgen.1008476.s022.tiff]

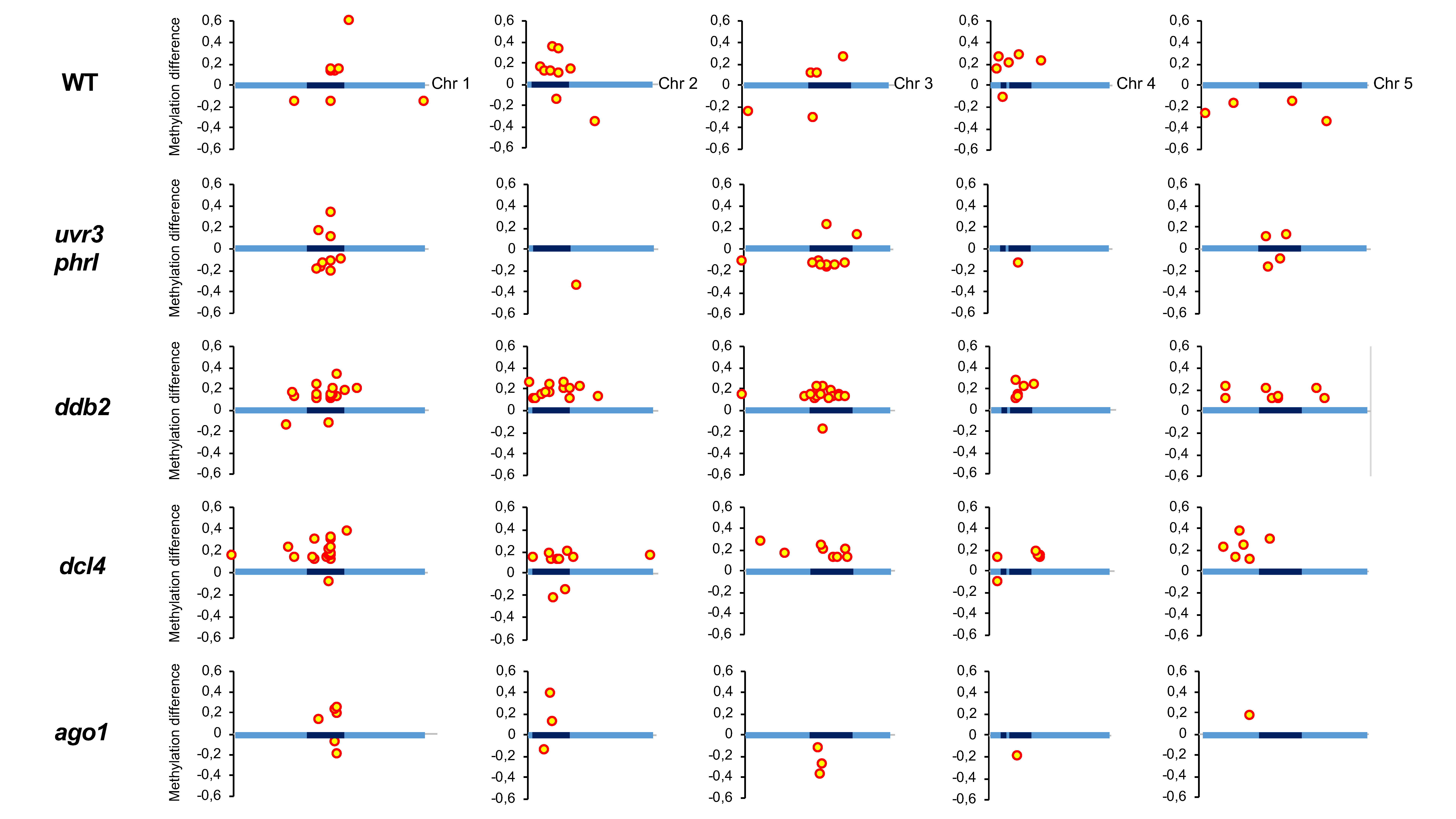

Supplement: S23 Fig — Distributions of DMRs overlapping (Stricto sensu) with photolesions along the Arabidopsis chromosomes (light blue: chromosome arms, dark blue: pericentromeric regions). Hyper- and hypo-DMRs are shown above and below each chromosome, respectively. (TIFF) [file pgen.1008476.s023.tiff]

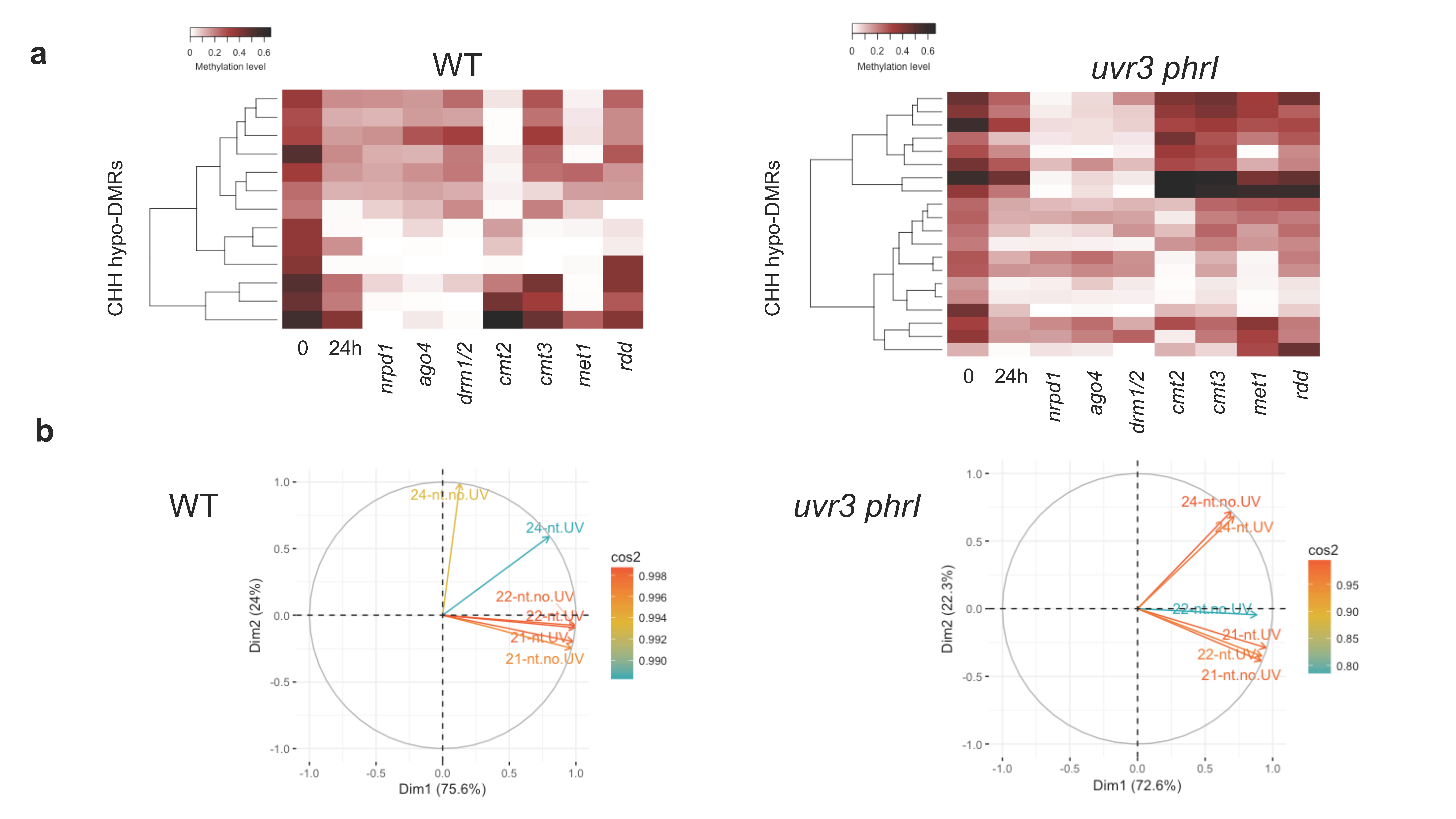

Supplement: S24 Fig — a Heatmaps of CHH methylation levels within hypo-DMRs identified in WT and uvr3 phrI plants before, 24h upon UV-C exposure. Columns represent data for each indicated genotype (white, 0; black, 0.6). b Circles of correlations between 21-, 22- and 24-nt small RNAs mapping to the hypo-DMRs overlapping with photolesions in WT and uvr3 phrI plants. (TIFF) [file pgen.1008476.s024.tiff]

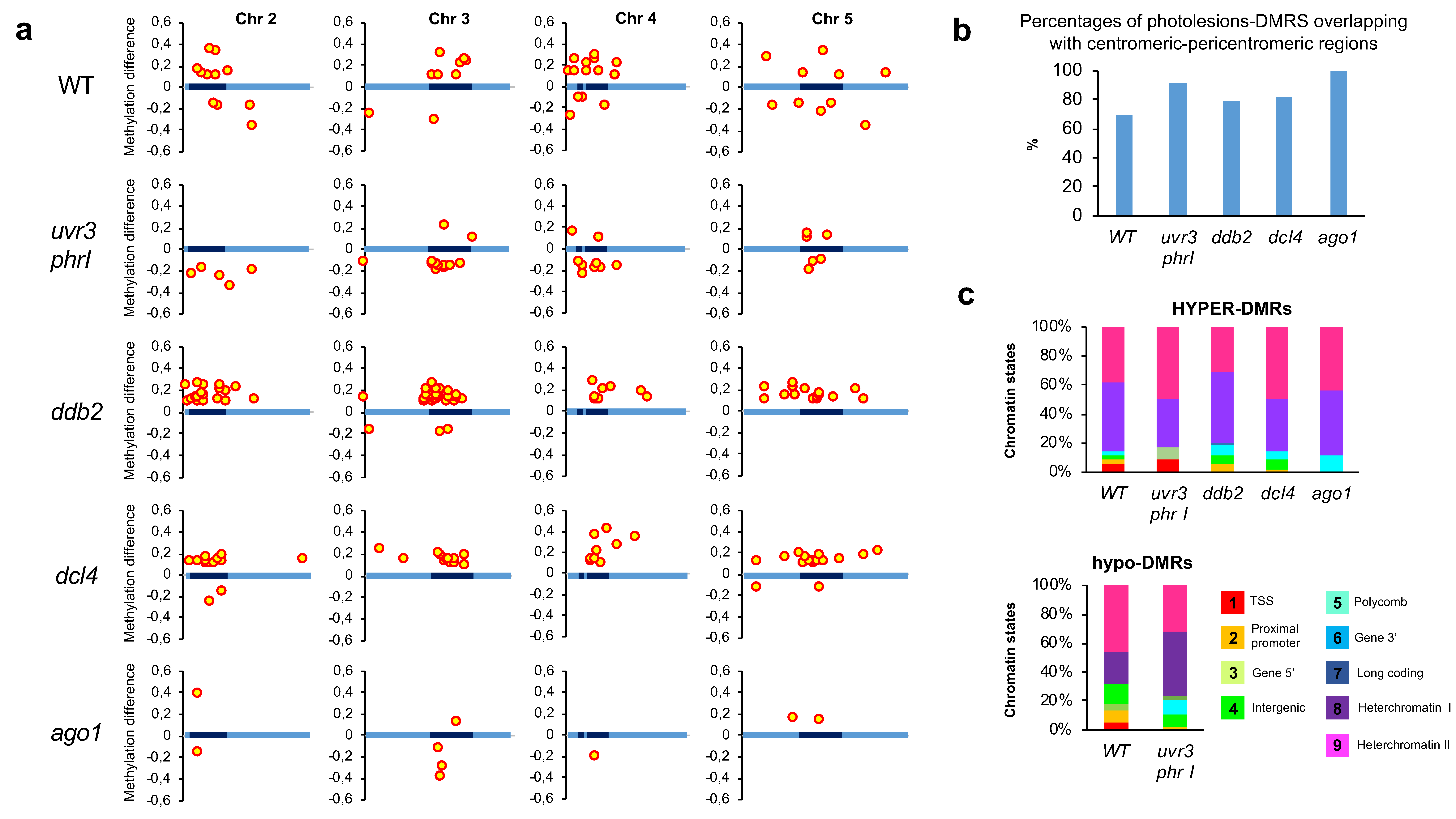

Supplement: S25 Fig — a Distributions of DMRs overlapping with photolesions along the arabidopsis chromosomes (light blue: chromosome arms, dark blue: pericentromeric regions). Hyper- and hypo-DMRs are shown above and below each chromosome, respectively. b Histogram representing the percentage of hyper- and hypo-DMRs overlapping with photolesions located within centromeric and pericentromeric regions. c Histograms representing the distribution of the 9 chromatin states of DMRs overlapping with photolesions. (TIFF) [file pgen.1008476.s025.tiff]

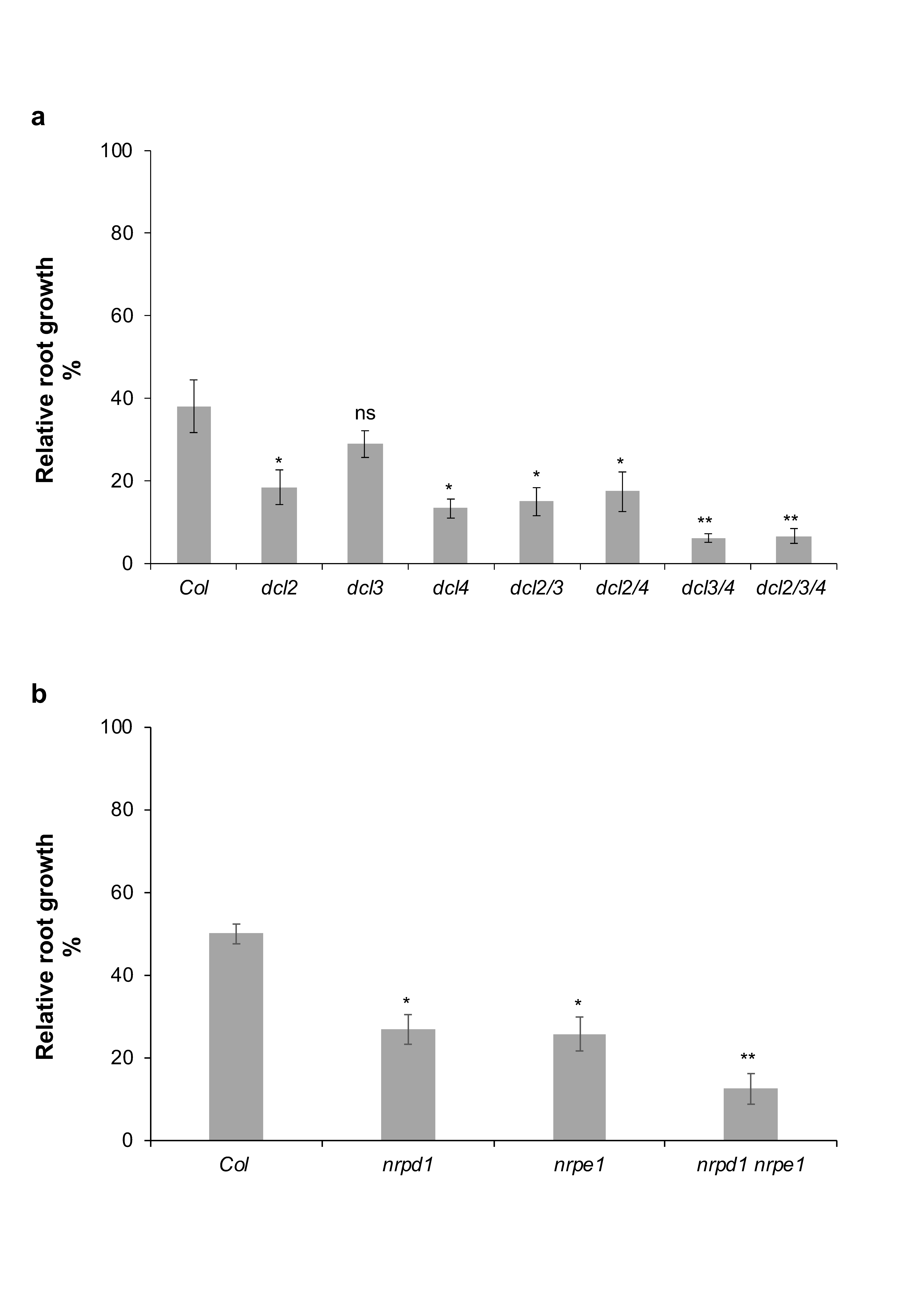

Supplement: S26 Fig — a Genetic interaction between dcl2, dcl3 and dcl4. Seven-day-old WT, single (dcl2, dcl3 and dcl4) and double (dcl2/3, dcl2/4, dcl3/4 and dcl2/3/4) mutant plants were exposed to UV-C (900 J/m2). Root growth was calculated relative to the corresponding untreated plants (±SD). Eight plants per replicate were used and experiments were triplicated. t-test *p<0.01 compared to WT; ** p<0.01 compared to dcl3 and dcl4; ns: non-significant. b Genetic interactions between nrpd1 and nrpe1. Seven-day-old WT, single (nrpd1 and nrpe1) and double (nrpd1nrpe1) mutant plants were exposed to UV-C (900 J/m2). t-test *p<0.01 compared to WT; ** p<0.01 compared to each single mutant. (TIFF) [file pgen.1008476.s026.tiff]
