# Supplementary material for: Photodamage repair pathways contribute to the accurate maintenance of the DNA methylome landscape upon UV exposure
Source: PLoS Genet. 2019 Nov 18;15(11):e1008476. doi: 10.1371/journal.pgen.1008476 (PMC6886878; doi:10.1371/journal.pgen.1008476)
Supplement: S1 Table — (DOCX) [file pgen.1008476.s027.docx]

**Supplemental Table 1 :** **Statistics of di-pyrimidines frequencies**

Di-pyrimidine frequencies for each DNA strand (+ and -) of damaged intergenic, TE and Protein Coding Genes (PCGs) regions were calculated and compared to di-pyrimidine frequencies of the Arabidopsis thaliana genome (TAIR10). Exact p values calculated according Mann Whitney test.

red: higher compared to the Arabidopsis genome

blue: lower compared to the Arabidopsis genome

black : non significant

|  | **WT** | ***uvr3 phrI*** | ***ddb2*** | ***dcl4*** | ***ago1*** |
| --- | --- | --- | --- | --- | --- |
| **CC+ inter** | 0.000002 | 4.108066e^-22^ | 1.043065e^-11^ | 1.047994e^-12^ | 0.073404 |
| **CC- inter** | 0.001245 | 1.034353e^-22^ | 1.181482e^-07^ | 2.386710e^-10^ | 0.726298 |
| **CT+ inter** | 0.002855 | 0.002043 | 0.430421 | 0.848763 | 0.773534 |
| **CT- inter** | 0.000021 | 0.005344 | 0.046338 | 0.375911 | 0.657247 |
| **TC+ inter** | 0.193070 | 9.911872e^-12^ | 0.000154 | 0.000057 | 0.006827 |
| **TC- inter** | 0.278745 | 4.827580e^-10^ | 0.555606 | 0.035694 | 0.315787 |
| **TT+ inter** | 2.437233e^-07^ | 0.378616 | 1.148263e^-08^ | 0.000384 | 0.000056 |
| **TT- inter** | 8.129112e^-08^ | 0.009999 | 0.012661 | 0.000759 | 0.230810 |
| **CC+ TE** | 0.000159 | 0.026875 | 0.678068 | 0.880010 | 0.034961 |
| **CC- TE** | 0.325881 | 1.127627e^-37^ | 0.000002 | 3.445125e^-15^ | 0.006277 |
| **CT+ TE** | 5.181050e^-20^ | 0.131896 | 0.009273 | 0.004097 | 0.001526 |
| **CT- TE** | 0.032106 | 3.569692e^-10^ | 0.054896 | 2.674007e^-07^ | 0.497429 |
| **TC+ TE** | 0.461676 | 4.164820e^-20^ | 0.000007 | 1.740909e^-12^ | 0.000724 |
| **TC- TE** | 0.140003 | 3.390645e^-19^ | 0.000216 | 8.813322e^-15^ | 0.080409 |
| **TT+ TE** | 5.371071e^-36^ | 1.553131e^-11^ | 2.518669e^-13^ | 4.612514e^-14^ | 1.599210e^-13^ |
| **TT- TE** | 1.429321e^-18^ | 0.000010 | 0.000022 | 0.110040 | 0.340116 |
| **CC+ PCG** | 1.598449e^-165^ | 2.764503e^-90^ | 1.025532e^-64^ | 7.759190e^-88^ | 1.410070e^-14^ |
| **CC- PCG** | 5.584896e^-159^ | 6.751455e^-68^ | 9.031742e^-51^ | 1.114985e^-87^ | 2.692716e^-07^ |
| **CT+ PCG** | 2.425401e^-07^ | 1.300389e^-29^ | 4.099648e^-10^ | 2.583149e^-19^ | 0.000010 |
| **CT- PCG** | 2.805784e^-12^ | 0.000316 | 0.000008 | 0.014012 | 0.005046 |
| **TC+ PCG** | 3.355920e^-34^ | 9.442238e^-50^ | 1.392673e^-24^ | 1.793070e^-30^ | 1.182933e^-11^ |
| **TC- PCG** | 1.466859e^-35^ | 3.508585e^-08^ | 3.041257e^-11^ | 3.126330e^-10^ | 0.000151 |
| **TT+ PCG** | 7.635052e^-15^ | 0.224611 | 0.096155 | 0.000001 | 2.044536e^-08^ |
| **tt- PCG** | 1.162764e^-18^ | 3.253269e^-09^ | 0.728806 | 2.391625e^-14^ | 0.003074 |
