# Supplementary material for: Photodamage repair pathways contribute to the accurate maintenance of the DNA methylome landscape upon UV exposure
Source: PLoS Genet. 2019 Nov 18;15(11):e1008476. doi: 10.1371/journal.pgen.1008476 (PMC6886878; doi:10.1371/journal.pgen.1008476)
Supplement: S2 Table — (DOCX) [file pgen.1008476.s028.docx]

**Supplemental Table 2:** Statistics of the high throughput sequencing experiments

Whole Genome Bisulfite Sequencing (WGBS)

| **Sample** | **Number of reads** | **Clean reads** | **Mapped reads** |
| --- | --- | --- | --- |
| WT 0 | 24 799 299 | 23 697 656 | 5 515 940 (23.3%) |
| *ddb2* 0 | 39 089 922 | 37 459 779 | 27 508 313 (73.4%) |
| *uvr3 phrI* 0 | 55 452 923 | 52 683 646 | 47 110 980 (89.4%) |
| *dcl4* 0 | 54 254 262 | 51 825 418 | 47 394 165 (91.4%) |
| *ago1* 0 | 50 337 222 | 48 156 436 | 39 258 943 (81.5%) |
| WT 24h | 52 347 786 | 49 702 705 | 45 580 743 (91.7%) |
| *ddb2* 24h | 53 297 047 | 51 321 223 | 45 627 472 (88.9%) |
| *uvr3 phrI* 24h | 46 756 341 | 44 973 274 | 40 559 204 (90.2%) |
| *dcl4* 24h | 52 542 646 | 50 125 786 | 46 528 825 (92.8%) |
| *ago1* 24h | 52 747 953 | 50 711 267 | 46 211 254 (91.1%) |

Immuno-Precipitation Of UV-damaged DNA (IPOUD)

| **Sample** | **Number of reads** | **Clean reads** | **Mapped reads** |
| --- | --- | --- | --- |
| input | 9 395 384 | 8 384 750 | 4 682 424 (55.8%) |
| WT 6,4 PP | 11 293 498 | 10 466 543 | 257 258 (2.5%) |
| WT CPD | 18 038 164 | 17 080 641 | 406 355 (2.4%) |
| *ddb2* 6,4 PP | 15 931 588 | 14 955 429 | 446 234 (3%) |
| *ddb2* CPD | 18 279 550 | 17 380 256 | 280 916 (1.6%) |
| *uvr3 phrI* 6,4 PP | 16 153 008 | 15 286 810 | 499 430 (3.3%) |
| *uvr3 phrI* CPD | 13 978 557 | 13 003 628 | 427 924 (3.3%) |
| *dcl4* 6,4 PP | 14 332 390 | 13 513 948 | 545 923 (4.0%) |
| *dcl4* CPD | 14 974 110 | 14 148 505 | 290 613 (2.0%) |
| *ago1* 6,4 PP | 13 300 629 | 12 388 736 | 193 769 (1.6%) |
| *ago1* CPD | 18 128 703 | 17 105 325 | 137 551 (0.8%) |

Small RNA-seq

| **Sample** | **Number of reads** | **15- to 41-nt reads** | **Mapped reads** |
| --- | --- | --- | --- |
| WT 0 | 19 677 898 | 14 992 007 | 12 695 174 (84.6%) |
| *ddb2* 0 | 21 989 130 | 17 235 777 | 14 532 863 (84.3%) |
| *uvr3 phrI* 0 | 19 640 581 | 15 254 723 | 12 585 739 (82.5%) |
| *dcl4* 0 | 22 671 494 | 16 281 510 | 13 022 642 (79.9%) |
| *ago1* 0 | 19 688 909 | 16 092 269 | 11 775 752 (73.1%) |
| WT 24h | 27 525 582 | 24 196 187 | 20 193 020 (83.4%) |
| *ddb2* 24h | 22 315 226 | 17 644 910 | 14 194 205 (80.4%) |
| *uvr3 phrI* 24h | 21 924 679 | 18 850 437 | 15 544 956 (82.4%) |
| *dcl4* 24h | 23 111 040 | 18 963 195 | 15 554 742 (82.0%) |
| *ago1* 24h | 18 586 123 | 12 437 522 | 10 194 498 (81.9%) |
