# Supplementary material for: Photodamage repair pathways contribute to the accurate maintenance of the DNA methylome landscape upon UV exposure
Source: PLoS Genet. 2019 Nov 18;15(11):e1008476. doi: 10.1371/journal.pgen.1008476 (PMC6886878; doi:10.1371/journal.pgen.1008476)
Supplement: S3 Table — (DOCX) [file pgen.1008476.s029.docx]

**Supplemental Table 3 :** Primers sequences used for qPCR

| **Name** | **Sequence** |
| --- | --- |
| MET1-fw | CGTGGTTCCAAAAGGAGATAAG |
| MET1-rv | TGTTTGGGAGACAAAAAGGAAT |
| DRM2-fw | TCGTTTCAGGTTGATACTGTGG |
| DRM2-rv | TGGTTAGTTTGCTCCCAAAAGT |
| CMT3-fw | AGGGTCAAACTTGAATCTGGAA |
| CMT3-rv | GATAAAACCCGATTTTGCTCTG |
| GAPDH -fw | TTGGTGACAACAGGTCAAGCA |
| GAPDH-rv | AAACTTGTCGCTCAATGCAAT |
| UbiCRed-fw | ACAAGCCAATTTTTGCTGAGC |
| UbiCRed-rv | ACAACAGTCCGAGTGTCATGGT |
| Hexo-fw | GGCGTTTTCTGATAGCGAAAA |
| Hexo-rv | ATGGATCAGGCATTGGAGCT |
| ROS1-fw | AAGGTCACATGTTGTGAACCAAT |
| ROS1-rv | ATGCTCGTCTGGAAGTTCGTA |
| RTPCR5S1 | GGATGCGATCATACCAG |
| 5SUNIV1 | CGAAAAGGTATCACATGCC |
| 180(all)-F | ACCATCAAAGCCTTGAGAAGCA |
| 180(all)-R | CCGTATGAGTCTTTGTCTTTGTATCTTCT |
| DML3-fw1 | GGACAATTCAGGATTCTTTCAGA |
| DML3-rv1 | CTTCACTAAATGTGATGGAGCATT |
| DML2-fw1 | TCTGTATCCTCGATTTGTAAAGGTT |
| DML2-rv1 | CCTTACACAGACATATCCTTCCTG |
| CMT2-fw | GATCACAGGCCGTTCCATATAA |
| CMT2-rv | ACAGTTTCATCCCACCAAAGA |
| COPIA78F_RT | CCACAAGAGGAACCAACGAA |
| COPIA78R_RT | TTCGATCATGGAAGACCGG |
