# Supplementary material for: Photodamage repair pathways contribute to the accurate maintenance of the DNA methylome landscape upon UV exposure
Source: PLoS Genet. 2019 Nov 18;15(11):e1008476. doi: 10.1371/journal.pgen.1008476 (PMC6886878; doi:10.1371/journal.pgen.1008476)
Supplement: Attachment — Submitted filename: Response to reviewers Graindorge et al .docx [file pgen.1008476.s030.docx]

We would like to thank the reviewers for their valuable and constructive comments.

We highlighted in red, the changes/corrections.

**Reviewer #1**

Line 307, for “UV-C irradiation induces chromocenters reshaping” , “induces” is better than “induced”. Line 215 et al.

We corrected as suggested.

In text, authors use Fig, but for lines 1098-1170, authors use Figure.

We corrected as suggested.

Lines 193 and 195, Figs are better.

We corrected all along the text as suggested.

Line 741, materials are better.

We corrected as suggested.

Lines 800 and 811, the untreated is better than untreated.

We corrected as suggested.

**Reviewer #2**

How many replicates were performed for this and how consistent was DMR identification between replicates?

As mentioned in the materials and methods section : „Leaves samples of two biological replicates were pooled for genome wide studies”. We decided to design our experiment in such way due to the limitation of our budget. We are aware that this pooling strategy neutralizes the most extreme outliers. However, it generates a more average signal. We believe that this strategy, combined with appropriate statistical analyzes robustly support our findings.

Presumably independent UV-C treatment would induce variable patterns of damage caused by stochastic exposure of different genome regions to radiation in different cells?

We agree with this comment. Each UV-C treatment may lead to an independent photodamage pattern due to irradiation of different cells/genomic regions. Therefore, we consider that each irradiation experiment leads to a particular photodamage profile, implying that genome wide studies related to photolesions must include CPD/6,4 PPs mapping.

At line 192 - are these DMRs changed in all sequence contexts, or just CG, CHG or CHH?

We clarified this point by providing more details in the text : „predominantly in the asymmetric CHH context”.

Lines 194-197 - this is a little confusing - H3K27me3 is typically not found in DNA methylated regions (at least in wild type), so it’s surprising to see this overlap reported.

Indeed, regions containing H3K27me3 is typically not found in DNA methylated regions. We rephrased this part to avoid any overinterpretation of the results: “…..overlapping with chromatin state 6 (intergenic regions) and with constitutive heterochromatic states 8 and 9 (GC rich within intergenic regions and TE; Fig. S1b)”

Could the authors provide more detail on how overlap analysis was performed?

We added a description of the overlap methodology in the materials and methods section.

Line 199-200 I am very unclear with what the authors mean by constitutive versus facultative heterochromatin - please explain this more clearly and show how overlap was assessed here.

We changed this sentence. We introduced the features of chromatin states 8 and 9 representative of constitutive heterochromatin.

Line 197 – I am also not clear what this statement about chromatin states and their overlap differing significantly from the genome average means - please explain.

Statistics have been applied to conclude about the significant difference of this distribution (lines 196 to 200). Details are given in figure legend S1b.

If the DMRs are found more frequently in heterochromatin does this not simply arise from the fact that heterochromatin has higher methylation levels?

We agree with this comment but we have to consider particular features of these DMRs.

Interestingly, the genomic regions gaining DNA methylation (hyper-DMRs), although located in the centromeric-pericentromeric area, are moderately methylated prior UV irradiation (Fig. S3A). It is likely that these regions are more prone to gain DNA methylation because (i) pathways (i.e. RdDM) already control their basal DNA methylation levels and/or (ii) pre-existing repressive chromatin marks, including DNA methylation, are flexible enough to be modulated upon UV-C exposure allowing gain or loss of DNA methylation.

In Figure 1 no information is presented on the reproducibility of these results - for each genotype how many replicates were performed and what correlation was observed between the changes?

As mentioned in the materials and methods section we used „Leaves samples of two biological replicates were pooled for genome wide studies”. Therefore correlation test cannot be applied.

Aside from plotting DMRs as points in 1B could the authors also simply plot the moving average of CG CHG and CHH methylation along the chromosome?

We decided to plot the DMRs because they have been calculated for exact coordinates and passed statistical test.

We believe that representing the average changes of CG, CHG or CHH along the chromosome will smooth the peaks of changes due to the low scale of display.

To what extent are total methylation levels changed? One might expect that CHH levels would show some difference from this way of looking?

The changes of CHH methylation levels are predominant and significant in all the tested samples based on our analytical method (Fig. 1c). As suggested, we included boxplot representing the CHH methylation differences identified in WT, *uvr3 phrI*, *ddb2*, *dcl4* and *ago1* plants in Fig. S2b as well as statisitics.

Line 232 onwards. The authors describe DMRs in several genotypes - what is the overlap between these DMRs between genotypes? Again replicate information is lacking.

We compared the overlap between WT and all the mutant plants as shown in Figs. S5 and S6.

We added in the same figures the comparisons in between genotypes.

On line 240 the authors mention that DDB2 has been connected with AGO4 - were ago4 mutants also tested in addition to ago1?

We did not perform a genome wide analysis of *ago4* because to our knowledge AGO4 does not act in the upstream steps of the photodamage repair process.

However, as you suggested and based on our findings it would be of interest to analyze *ago4* as well the double *ddb2 ago4* mutant plants upon UV-C exposure to decipher the role of the canonical RdDM in the gain of DNA methylation.

Line 241-242 I don't agree with this sentence - how do the detection of DMRs in ddb2 support a role for small RNAs?

To avoid any over-interpretation and confusion we rephrased the sentence. We highlighted that our data “suggest that the small RNA mediated DNA repair pathway, involving DDB2 and DCL4, also controls DNA methylation”.

The authors again mention DMR overlap with TEs - the authors need to perform a test whether this is significantly more than expected, especially as TEs and methylation and strongly correlated in the first instance.

We thank the reviewer for this comment. To gain in robustness, we added in the graph the distribution of PCG, TE and intergenic regions in the Arabidopsis genome (*A. t*). We performed a statistical test that supports our conclusions.

The size of DMRs is described as being changed in the mutants compared to wild type - please state what the difference is in the text - is this change significant?

The exact p value are already shown in Fig. S3a. Now, we stressed the point that these differences are significant.

The authors propose that this reflects a change in spreading - this seems poorly supported by the presented data.

We agree with this comment. We rephrased the sentence : “This suggests that each DNA repair process restricts the length of regions exhibiting DNA methylation alterations”.

It would be useful if the authors could provide screenshots from browsers (including replicate samples) showing clear examples of DMRs in each case, in addition to examples showing the stated change in spreading of methylation.

We used, in Fig. S4, the *ONSEN* locus that shows a clear example of DMRs sizes in all tested samples. Following your suggestion, we added genome browser views in Fig. S3b.

The trends of DNA methylation alterations as well as the DMRs sizes are representative of the take home message of our analyzes.

As phenotypes are observed with dcl4, have the authors also tested rdr6, which shares phenotypes and siRNA changes with dcl4?

We agree with this comment. However, we did not check *rdr6* because we found synergistic interactions with both *ddb2* and *dcl4* during UV-C response (Schalk et al., 2017) suggesting that under such stress, RDR6 acts in another pathway. However, we cannot exclude that
discrete loci may be under the control of RDR6. This could be analyzed in the future.

In Figure 2a could the authors provide a clearer example of what ‘% chromocenter occupancy’ represents? The images in part b do not present a very convincing case for meaningful changes. Based on the images shown it is very hard to conclude that there are any changes here. Perhaps measurement in mutants such as ddm1 or met1 would yield a more convincing change to calibrate against?

We better defined what chromocenter occupancy (CO) is (lines 320-321).

Pictures are representative of the median CO for each genotype. Indeed it is difficult to see by eyes the differences. The aim of developing a computer based analysis was to overcome this problem. Using large amount of nuclei-chromocenters and statistical analysis allow concluding whether significant differences between samples exist or not.

As you mentioned, we trained the program using *ddm1* mutant plants. Please find below, how such assay was performed as well as the corresponding set of data.

Line 201. Please explain more clearly how the conclusion that CHH sites are most changed has been reached?

As mentioned in the materials and methods (section DMRs Calling) this conclusion is based on the DMR calling procedure for each context including methylation changes thresholds (CG > 0.4; CHG > 0.2 and CHH > 0.1 and p value (< 0.05 according Wilcoxon test).

Is the 97% referring to DMRs, or to specific cytosines that have been identified as changed.

The 97% changes refer to the CHH context as already mentioned in the text.

Lines 298-300: CHH patterns between several of the mutants analyzed are described as ‘rather resembling’ - what does this mean? Please try and be more quantitative and rigorous in your analysis and description of results.

We thank you for this comment. We provided a more quantitative analysis using boxplot and statistics. We rephrased the sentence and rewrote the corresponding figure legend.

Line 213. Again I am very confused about what the authors are defining as facultative versus constitutive heterochromatin? This distinction is made throughout the paper with no explanation of how these sequences are defined.

We removed this terminology and added “centromeric-pericentromeric regions representing constitutive heterochromatin”.

Line 264-265. What does this statement mean? How are the TEs in question ‘over-represented compared to their distribution’ - please provide a clearer explanation of how this is tested for. Also Class I TEs encompass more types of TE than just LTR/Gypsy. Do the authors mean all RNA TEs here, or just Gypsy?

In order to clarify our meaning we rephrased the sentence and detailed in the figure legend the statistical analysis used to support these trends.

Again it would be beneficial to the reader to have the BS-seq data presented in a browser at loci of interest that represent clearly the types of changes reported via genome-wide analyses.

We used, CIRCOS showing the location of the DMRs in each sample (Fig. S2). Given that only few DMRs overlap in between samples, it was difficult to provide a single representative screen shot. However, we provided such data display for the *ONSEN* locus where DMRs can be identified in each sample (Fig. S4a).

The authors invoke UV-C induced changes to DNA methylation as a TE defense mechanism. However, no attempt is made to test for increased transposition after UV-C exposure in the mutants tested - this should be performed if they want to propose this.

Indeed we suggest that “….UV-C irradiation may have transiently released TEs expression and that changes in DNA methylation could act as a defense mechanism….”.

We were very careful in our sentence. You can notice that we used the word “mobilization” that is the first step of transposition. This TE mobilization may activate well characterized PTGS-TGS processes to repress the full transposition process (mobilization + integration).

We are interested in deciphering such UV-C-dependent process. It would imply to perform RNA seq + mobilome experiments during a time course including early time points (30 min, 1h, 2h upon UV-C exposure) and also a full genome sequencing using Nanopore technology at latest time points (24h to 48h upon UV-C exposure). These combined analyses would allow quantifying TE mobilization and identification of their new insertion sites, if any. We believe that this set of experiments belong to another story for which a substantial budget must be dedicated.

The authors continue to analyze siRNA patterns around the identified DMRs and observe various correlations. I am not clear how these relate to the original hypothesis? Have the authors sequenced siRNAs during the UV-C response?

The original hypothesis was to test whether changes of DNA methylation (gain or loss) are correlated with alterations of small RNA abundance, mainly 24-nt siRNA. Given that siRNA originating from POL IV dependent loci are also involved in small RNA-mediated repair of photolesions (21-nt siRNA) we aimed at testing the effect of the UV-C irradiation on this particular repair process and on RdDM.

Importantly, in the present study we sequenced small RNA populations prior and 24h upon UV-C exposure. In Schalk et al. (2017) we analyzed the small RNA populations prior and 30 min upon UV exposure. We used these publicly available data to recalculate the global 24-nt siRNA abundance at 30 min and found a significant increase compared to time point 0 (Fig. S13b).

The descriptions used are also imprecise and opaque in meaning at many points - for example line 428 ‘siRNA patterns look much more complex’ - what does this mean? How is complexity assessed?

We agree we this comment. We rephrased this part of the paragraph to be more precise.

An interesting approach is made to map DNA damage by using ChIP with CPD and 6,4-PP antibodies. However, again the authors provide no information on replicates and how consistent the patterns observed are between independent replicates.

As mentioned in the materials and methods section we used „Leaves samples of two biological replicates were pooled for genome wide studies”.

Importantly the quality of the irradiation and of the IPOUD (Immuno-Precipitation Of UV Damaged DNA) experiments have been checked using internal control called “hot spots of photodamage” (Schalk et al., 2017) that correspond to genomic regions exhibiting significant enrichment of photolesions in all experiments conducted in the group since 5 years (IPOUD-NGS and IPOUD-qPCR with more than15 independent experiments). In the present study we recovered 20/23 hot spots, in all tested plants, supporting the idea that both irradiation and IPOUD have successfully worked.

Please find below 6 examples of hot spots retrieved from our IPOUD experiments.

Is it possible to repeat this experiment using different UV exposures to confirm that the signal increases with higher levels of damage?

Yes, it would be interesting to perform such experiment. The only limitation would be the budget to determine the genomic locations of photodamage.

Importantly, the UV-C dose used in this study was set up to induce photolesions and also to prevent significant changes in developmental phenotypes in DNA repair defective mutant plants.

On lines 453-454 - what mutants are being referred to here? Please be more specific? Which mutants were tested and why?

We added in the paragraph that the number of regions enriched in photolesions are much more reduced in all tested mutant plants compared to WT plants (lines 458-459).

Why would photolesion enrichment go down in these mutants (whichever they are?)?

We already explained, based on published data, the reason why less regions have been identified in mutant plants compared to WT plants: “This could be due to the physiological adaptation of DNA repair deficient plants which are prone to produce more UV-screen compounds to prevent excessive photo-damage and also to the reduced leaves size of the *ago1-27* plants”.

Lines 456-458 - this is very interesting potentially - so TEs/intergenic regions are more prone to damage? This could represent (i) higher dimer formation in heterochromatin, (ii) reduced repair in heterochromatin, (iii) increased repair in euchromatin - or all/a combination of these factors - which do the authors think is occurring?

Our data reflect a higher formation of photolesions because we harvested the samples immediately upon UV-C exposure. To further decipher the repair efficiency of euchromatin *vs* heterochromatin, as suggested, a kinetics would be more appropriate using different combination of mutant plants (i.e. WT *vs ddb2* with samples harvested immediately upon UV-C exposure, 1h, 2h, 6h and 24h upon UV-C exposure).

Line 459 - the authors state here that they think that heterochromatin has ‘high reactivity’ please clarify your thoughts here?

We clarified our thoughts by changing the sentence to : “….this part of the genome is more prone to form photodamage” (lines 464-465).

What does higher reactivity mean at the molecular level? Would you expect any of your mutants tested to change this?

Higher reactivity means higher enrichment of photolesions at particular genomic regions. In order to test this it would also be interesting to map the photolesions in mutant plants exhibiting reduced DNA methylation levels such as DNA methyltransferases defective plants (*met1*, *cmt2*, *cmt3,* *drm2*) or *rddm* mutant plants. Additionally mutant plants affected in histone methyltransferases/demethylases expression (*kyp*, *atxr5/6*, *ibm1*…) would also be a materiel of choice to test the level of photolesions enrichment.

*ddb2* and active DNA methylation mutant plants (i.e. *rdd*) could also be used to test whether higher DNA methylation levels lead to different photodamaged profile.

It would be helpful to see a browser track showing the ChIP DNA samples (and replicates) in enriched versus non-enriched regions?

The CIRCOS (Fig. S17) shows the fold enrichment (IP/input).

As mentioned in the materials and methods section : „Leaves samples of two biological replicates were pooled for genome wide studies”, therefore replicates cannot be displayed in the corresponding figure.

Perhaps a region that contains both expressed genes and heterochromatic TEs - can the difference in signal described be observed in such a way?

This suggestion is interesting. However, we have to keep in mind that the di-pyrimidines contents must be similar between these regions to allow a proper comparison and to avoid a bias in the analyzes.

Lines 467-470 - please explain more about these chromatin states - what are they? Which marks are they typified by? What biological roles do they play in the genome?

We detailed in the text the features of the cited chromatin states (lines 472-476).

Lines 470 - all mutants are said to ‘behave like WT’ - please explain - you mean the lesion enriched sites overlap the same places? (although they are fewer?) - but with a ‘more pronounced effect’ - so they are not the same then? Please clarify your meaning here.

We rephrased the sentence to clarify our meaning.

It is important to notice that although photolesions overlap with similar chromatin states in between samples, it does not imply that these photodamaged regions correspond to the same genomic coordinates *stricto sensu*.

Generally the reproductibility of the approach is an interesting question.

At Line 499 the authors compare photolesion ChIP-seq data with the loci that lose methylation after UV-C exposure. Were the photolesion and BS-seq data performed on the same tissue? This again comes back to replication - how many times has each experiment been repeated and how reproducible are the signals?

The same samples have been used for WGBS and IPOUD.

As mentioned in the materials and methods section : „Leaves samples of two biological replicates were pooled for genome wide studies”. Our statistical analyses reveal that the overlaps do not occur by chance.

Lines 504-505 1% of damaged regions show a change in DNA methylation (numerically how many places are being referred to here?).

We rephrased the sentence to give more details, including the exact number of overlapping sites for WT plants (line 509).

It is stated that the ‘DNA repair defective’ plants show a 2% change - is this significant? Which defective mutants are specifically being referred to?

We rephrased the sentence to specify which defective plants exhibit such enhancement compared to WT plants.

**Minor comments**:

Abstract line 29 - please clarify the use of ‘asymmetric’ here - do you mean CHH sites?

We clarified this point.

Abstract line 38 - please provide a more explicit definition of constitutive and facultative

heterochromatin.

We gave details about the chromatin states and used preferentially the terminology “repressive chromatin”.

Line 125 - Perhaps add ‘The’ to the start of this sentence.

We corrected as suggested.

Line 127. Please change to ‘inefficient’.

We corrected as suggested.

Line 131. Please change ‘prevents’ to ‘prevent’.

We corrected as suggested.

Line 136 - please change to ‘the DNA methylation landscape’.

We corrected as suggested.

Line 139 - formatting error of reference 23.

We corrected as suggested.

Line 143. Change ‘exists’ to ‘exist’.

We corrected as suggested.

Lines 219 and 463. Arabidopsis should have a capital letter.

We corrected as suggested.

**Reviewer #3**

I would suggest the authors to shorten the text, especially in the results and but also the discussion section concentrating on their most important findings and conclusions. This would make the whole story much more digestible also to the general reader.

We thank the reviewer for this suggestion.

We try to better focus on the findings in both results and discussion section.
