# Supplementary material for: Photodamage repair pathways contribute to the accurate maintenance of the DNA methylome landscape upon UV exposure
Source: PLoS Genet. 2019 Nov 18;15(11):e1008476. doi: 10.1371/journal.pgen.1008476 (PMC6886878; doi:10.1371/journal.pgen.1008476)
Supplement: Attachment — Submitted filename: Response to reviewer Graindorge et al revised 2.docx [file pgen.1008476.s031.docx]

Reviewer#2

First the question of correlation between your replicates. Please answer my question directly. For the replicates what is the correlation value between them? In the response the authors state that ‘two biological replicates were pooled… therefore correlation test cannot be applied’. Im afraid that doesn’t make sense - two samples can easily be correlated. Please answer the question.

As detailed in Materials and Methods (section UV-C treatment) of the original and of the revised version I of the manuscript : “**Leaves samples** of **two biological replicates** were pooled for genome wide studies”. In other words, the **plant material** **was mixed** of **two independent biological replicates** to perform **one DNA** and **one small RNA** **extraction per genotype** and **per time point**. Thus, one small RNA extract representing these two independent biological replicates was sequenced and one DNA extract representing these two independent biological replicates was treated with bisulfite, and immunoprecipitated with anti-photolesions antibodies and send for high throughput sequencing. Therefore, and as mentioned in the previous response, we cannot provide a correlation test because only **one set of genome wide** **data is available** for each genotype and for each time point.

This pooling strategy neutralizes the most extreme outliers but generates a more average signal. We believe that such approach was a realistic balance between well established/recognized approach (pooling strategy) and our budget limitation. For example such design has been used in Noshay et al. Plos Genetics 2019 to determine DNA methylation profile by WGBS (pooling of 3 biological replicates).

Importantly, the cross comparisons of the tested parameters (DMRs-small RNA-photolesions), supported by robust statistical tests, demonstrate the significance of the identified overlaps.

The authors are resistant to plotting methylation along the chromosomes as requested, instead focusing on DMRs. DMRs are fine, but I don’t see why methylation could not be plotted in the requested way. If it doesn’t look any different then that provides a clear indication of the magnitude of the effects at a genome wide scale.

We perfectly understand this point and we have now provided a new figure (called Fig. S3 in this revised version II) showing along the 5 chromosomes the CHH methylation levels prior (time point 0) and 24h upon UV-C exposure.

We would like to stress the point that our aim was to provide the most didactic display of our data. Given that we focused on methylation differences (DMRs) we thought that it was a didactic way to highlight changes. On the other hand, all the raw and the processed data have been deposited to NCBI and will be publicly available, as soon as the manuscript will accepted, allowing all sort of supplemental analyses and displays. Moreover, and in the interest of transparency, bedgraph, big wig files (from WGBS, IPOUD and small RNA experiments) are available on demand, to display data using easy and friendly genome browsers (i.e. IGV).

For one of my points I requested that the authors provide screenshots of their data shown in a genome browser. They provide for example in Figure S4 a plot around the ONSEN transposon, which is supposed to represent the trends reported genome wide. On this plot is highlighted DMRs by red rectangles. I want to see the data that the DMR is based on - I would suggest plotting the methyl value for each cytosine across this region as a histogram, i.e. the data that the DMR locations is based on. Equally, the siRNA data shown do not seem to be changing between the genotypes shown or between the time points in any of the single genotypes. I'm afraid if this is the ‘best’ locus the authors can provide it doesn’t fill me with a great deal of confidence about the data that underlies the conclusions being drawn.

We now provide a browser view of the *ONSEN* locus using IGV (called Fig. S5a in this revised version II). A new figure (Fig. S6) also shows several examples of DMRs overlapping with TE and in between genic regions or TE, to better illustrate our findings. Again, all bed graph files are available on demand and raw/processed data will be publicly available (GEO registration number : GSE132750) as soon as the manuscript will be accepted for publication.

Importantly, we would like to stress the point that we never claimed in all our versions (original and revised) that *ONSEN* was the best representative locus and that the overlapping 24-siRNA abundances changed between time points (lines 275-276). We used *ONSEN* as example, because it was identified as a heat responsive TE and we found interesting to describe its behavior upon UV-C exposure, given that light stress (damaging effect) and heat stress are closely related in global warming (please see line 709 in the discussion).
